# miR-3188 regulates nasopharyngeal carcinoma proliferation and chemosensitivity through a FOXO1-modulated positive feedback loop with mTOR–p-PI3K/AKT-c-JUN

**DOI:** 10.1038/ncomms11309

**Published:** 2016-04-20

**Authors:** Mengyang Zhao, Rongcheng Luo, Yiyi Liu, Linyuan Gao, Zhaojian Fu, Qiaofen Fu, Xiaojun Luo, Yiyu Chen, Xiaojie Deng, Zixi Liang, Xin Li, Chao Cheng, Zhen Liu, Weiyi Fang

**Affiliations:** 1 Cancer Center, Traditional Chinese Medicine-Integrated Hospital, Southern Medical University, Guangzhou 510315, China; 2 Cancer Research Institute, Southern Medical University, Guangzhou 510515, China; 3 Department of Pathology, Basic School of Guangzhou Medical University, Guangzhou 510182, China

## Abstract

The biological role of miR-3188 has not yet been reported in the context of cancer. In this study, we observe that miR-3188 not only reduces cell-cycle transition and proliferation, but also significantly prolongs the survival time of tumour-bearing mice as well as sensitizes cells to 5-FU. Mechanistic analyses indicate that miR-3188 directly targets mTOR to inactivate p-PI3K/p-AKT/c-JUN and induces its own expression. This feedback loop further suppresses cell-cycle signalling through the p-PI3K/p-AKT/p-mTOR pathway. Interestingly, we also observe that miR-3188 direct targeting of mTOR is mediated by FOXO1 suppression of p-PI3K/p-AKT/c-JUN signalling. In clinical samples, reduced miR-3188 is an unfavourable factor and negatively correlates with mTOR and c-JUN levels but positively correlates with FOXO1 expression. Our studies demonstrate that as a tumour suppressor, miR-3188 directly targets mTOR to stimulate its own expression and participates in FOXO1-mediated repression of cell growth, tumorigenesis and NPC chemotherapy resistance.

## Abstract (web summary)

Although miR-related mechanisms have been implicated in nasopharyngeal carcinoma (NPC), a precise role for miR-3188 has not been reported in this context. Here, Zhao *et al*. show that FOXO1-induced miR-3188 acts as a tumour suppressor in NPC by regulating the axis mTOR/PI3K/Akt/c-Jun.

## Graphical abstract

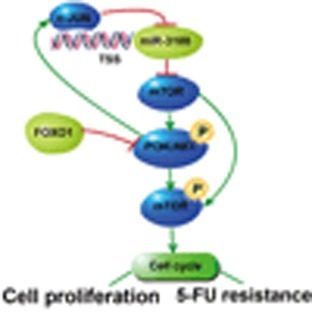

MicroRNAs (miRNAs or miRs) play important roles in development, cellular differentiation, proliferation, cell-cycle control and cell death^1^, and have been implicated in a variety of human diseases, including cancer^2^,^3^. A growing body of evidence has demonstrated the importance of miRNAs in managing chemotherapy efficacy in multiple human cancers^4^,^5^. Despite being one of the original miRNAs discovered, the biological role of miR-3188 and its molecular mechanisms underlying cancer initiation and progression have not been reported.

Nasopharyngeal carcinoma (NPC) is a tumour type arising from the epithelial cells that line the nasopharynx^6^. It is common in certain regions of East Asia and Africa, with Epstein-Barr virus (EBV) exposure, diet and genetic factors implicated in its aetiology^7^,^8^. Although relatively rare in the USA, NPC accounts for one-third of childhood nasopharyngeal neoplasms^9^. In recent studies, abnormal expression of miRNAs was been broadly implicated in the pathogenesis of NPC. For example, EBV-encoded miRNA BART1 induces tumour metastasis by regulating the PTEN-dependent pathways^10^. In addition, tumour suppressor PDCD4 modulates miR-184-mediated direct suppression of c-MYC and BCL2-blocking cell growth and survival^11^.

FOXO1 is a transcription factor and a member of the FOXO subfamily of the Forkhead/winged helix family. The phosphorylation of FOXO1 by AKT leads to its inactivation after nuclear to cytoplasmic translocation^12^,^13^. Previous evidence has supported that FOXO1 functions as tumour suppressor on the basis of its role in regulating cell-cycle progression, differentiation, metabolism and survival^14^,^15^. Furthermore, decreased FOXO1 expression has been demonstrated in many tumour types, such as Hodgkin lymphoma^16^, breast cancer^17^ and alveolar rhabdomyosarcoma^18^. Recent evidence suggested that LMP1 silencing slows cell growth and enhances chemosensitivity through inhibition of the AKT signalling pathway and its downstream factor phospho-FOXO1 in EBV-positive NPC cell line^19^. Elevated levels of phosphorylated AKT also correlated with phospho-FOXO1 in NPC samples^20^. However, the detailed role of FOXO1 in the suppression of NPC cell growth remains unclear.

Here, we examined the relationship between miR-3188, mammalian target of rapamycin (mTOR) and FOXO1 in NPC, and found an atypical miR-3188-mTOR–p-PI3K/AKT-c-JUN feedback loop modulated by FOXO1. This pathway suppresses proliferation and sensitizes NPC cells to 5-fluorouracil (5-FU). Altogether these results provide a mechanism by which miR-3188 modulates NPC cell growth.

## Results

### miR-3188 suppresses cell growth and 5-FU chemoresistance

**Figure 1:**
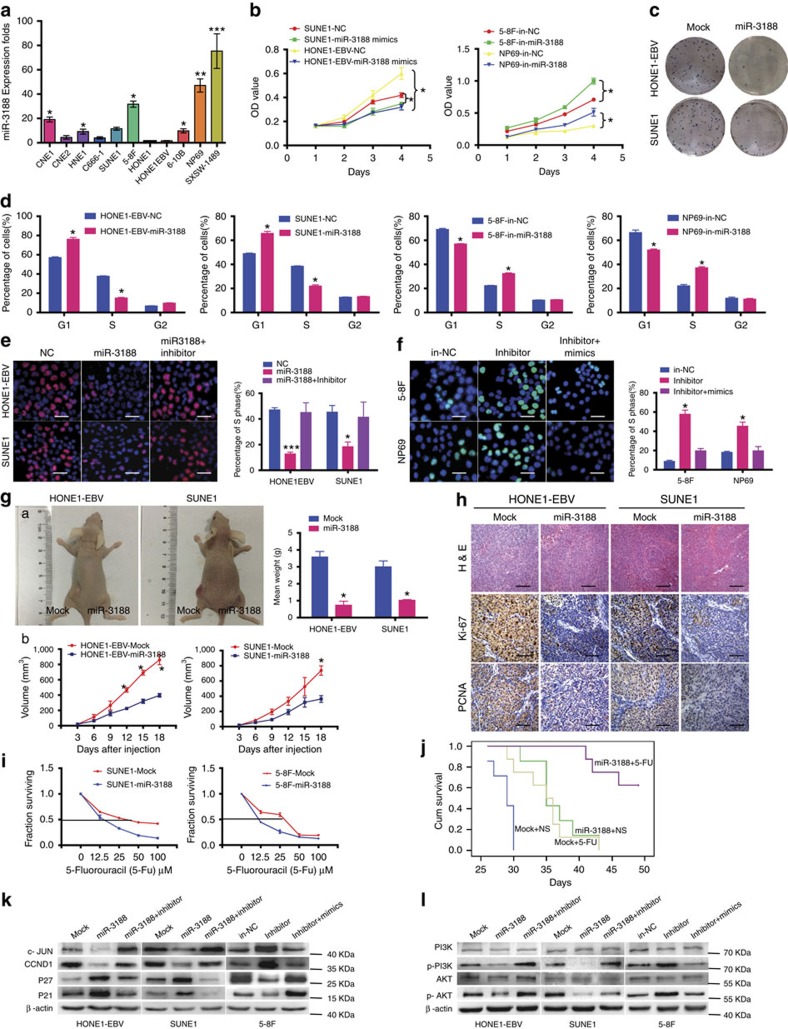
miR-3188 attenuates the growth of NPC cells *in vitro* and *in vivo* by inactivating PI3K/AKT. (**a**) qRT-PCR analysis of miR-3188 expression in NPC cell lines and immortalized human nasopharyngeaepithelial cell lines. One-way ANOVA and Dunnett's multiple comparison test. Mean±s.d., *P<0.05; **P<0.01; ***P<0.001. MTT assays (**b**), colony formation assay (**c**), FACS assays (**d**) and EdU incorporation assays (**e**,**f**) of NPC cells and NP69 were performed after transfection with NC, in NC and/or miR-3188 mimics, inhibitor as indicated. Scale bar: 15 μm. Parametric generalized linear model with random effects, Student's *t*-test, One-way ANOVA and Dunnett's multiple comparison test. Mean±s.d., *P<0.05; **P<0.01; ***P<0.001. (**g**) (a) The *in vivo* effect of miR-3188 was evaluated in xenograft mouse models bearing tumours originating from HONE1-EBV and SUNE1 cells, *n*=5/group; (b) tumour volume was periodically measured for each mouse and tumour growth curves was plotted. Parametric generalized linear model with random effects, *P<0.05. (**h**) Representative H&E as well as Ki67 and PCNA IHC stainings of primary tumour tissues are shown. Scale bar: 30 μm. (**i**) Dose-response curves of SUNE1 and 5-8F treated with miR-mock or miR-3188 48 h after treatment with 5-FU. Parametric generalized linear model with random effects. (**J**) Survival analysis showed cumulative overall survival time ranked low to high, as follows: Mock+NS<Mock+5-FU<miR-3188+NS<miR-3188+5-FU, *n*=9/group. Log-rank test. (**k**,**l**) Expression of PI3K, AKT, p-PI3K, p-AKT, c-JUN, CCND1, p27 and p21 were dected following 100 nmol transfection of miR-3188 inhibitor or mimics in SUNE1, HONE1-EBV and 5-8F cells. β-actin was used as a loading control.

To identify the role of miR-3188 in NPC development, we first examined its expression levels in normal epithelium (NP) and NPC cell lines. miR-3188 expression was elevated in NP69 and SXSW-1489 cells but weakly expressed in NPC cells (Fig. 1a). To further explore its biological role in NPC, miR-3188 mimics or inhibitors were respectively introduced into NPC or NP69 cell lines. More than threefold increase in miR-3188 expression was observed in HONE1-EBV and SUNE1 cells treated with miR-3188 mimics compared with the control group by qRT-PCR (Student's *t*-test, with *P*<0.05 for both) (Supplementary Fig. 1A). Due to higher endogenous miR-3188 expression in NP69 and 5-8F cells, miR-3188 inhibitors were transiently transfected into these lines. Expression of miR-3188 was significantly lower in the inhibitor-treated NP69 and 5-8F cells than in the control cells (Supplementary Fig. 1B, Student's *t*-test, *P*<0.05 for both).

Subsequently, we examined the effect of miR-3188 expression on NPC cells or NP cell growth *in vitro*. Using 3-(4,5-Dimethylthiazol-2-yl)-2,5-diphenyltetrazolium bromide (MTT) assay (Fig. 1b), colony formation (Fig. 1c and Supplementary Fig. 1C), cell-cycle analysis (Fig. 1d) and Edu incorporation assays (Fig. 1e), we found that overexpressed miR-3188 significantly suppressed cell growth and G1 to S cell-cycle transition in HONE1-EBV and SUNE1 cells. Conversely, suppression of miR-3188 markedly restored cell proliferation and induced G1/S transition in NP69 and 5-8F cells (Fig. 1b,d,f).

Next, we conducted an *in vivo* tumour formation experiment by subcutaneously injecting HONE1-EBV-miR-3188 and SUNE1-miR-3188 or control cells (Supplementary Fig. 1D) into nude mice. After 18 days of implantation, the mice injected with HONE1-EBV-miR-3188 and SUNE1-miR-3188 cells had smaller tumour burdens (Fig. 1g) and displayed lower expression of Ki67 and proliferating cell nuclear antigen (PCNA) in tumour tissues relative to controls (Fig. 1h). These results suggested miR-3188 significantly inhibits tumorigenesis *in vivo*.

NPC cell lines stably overexpressing miR-3188 exhibited significantly increased sensitivity to 5-FU. Inhibition rates 48 h after treatment at different concentrations of 5-FU were calculated for cells with or without miR-3188 transfection (Fig. 1i). The IC50 for 5-FU in SUNE1 cells was reduced from 42 to 14 μM after miR-3188 transfection. A similar IC50 reduction from 23 to 8 μM occurred in 5-8F cells. Interestingly, obvious changes in the IC50 for diamminedichloroplatinum (DDP) treatment was not observed in miR-3188-treated NPC cells (Supplementary Fig. 1E).

We then evaluated the *in vivo* anti-tumour efficacy of 5-FU in mice bearing tumours originating from miR-3188-overexpressing cells or their control lines. The weight of each group were measured every 3 days, and the results showed that tumour burden in mock+5-FU and miR-3188+5-FU groups was slightly reduced compared to those in mock+NS and miR-3188+NS groups, but there were no significant difference among the four groups (Supplementary Fig. 1F). This suggested that 5-FU was well tolerated by the mice. Kaplan–Meier analysis showed the survival times mice in the mock+5-FU and miR-3188+NS groups were much longer than the mock+NS group. However, the survival time of miR-3188+5-FU treatment group was significantly longer than the other three groups (Fig. 1j) (log-rank test, *P*<0.001). There was no significant difference between the mock+5-FU group and mock+miR-3188 groups. Average survival times of the mock+NS, miR-3188+NS, mock+5-FU and miR-3188+5-FU groups were 28.7, 36.4, 37.0 and 49.0 days, respectively.

To explore the mechanisms by which miR-3188 suppresses NPC cell proliferation, we found that miR-3188 overexpression downregulated c-JUN and CCND1 but enhanced p27 and p21. miR-3188 inhibitors rescued these decreased levels. Interestingly, miR-3188 knock-down in 5-8F cells exhibited opposite results and miR-3188 mimics could restore levels of these cell-cycle regulators (Fig. 1k). Furthermore, we found levels of p-PI3K and p-AKT were decreased in miR-3188-overexpressing SUNE1 and HONE1-EBV cells, yet increased in miR-3188-inhibited 5-8F cells (Fig. 1l). These results suggest that miR-3188 decreases cell growth by inactivating phosphoinositide-3-kinase (PI3K)/AKT as well as downstream c-JUN and G1/S cell-cycle transition signalling.

### miR-3188 directly targets mTOR

**Figure 2:**
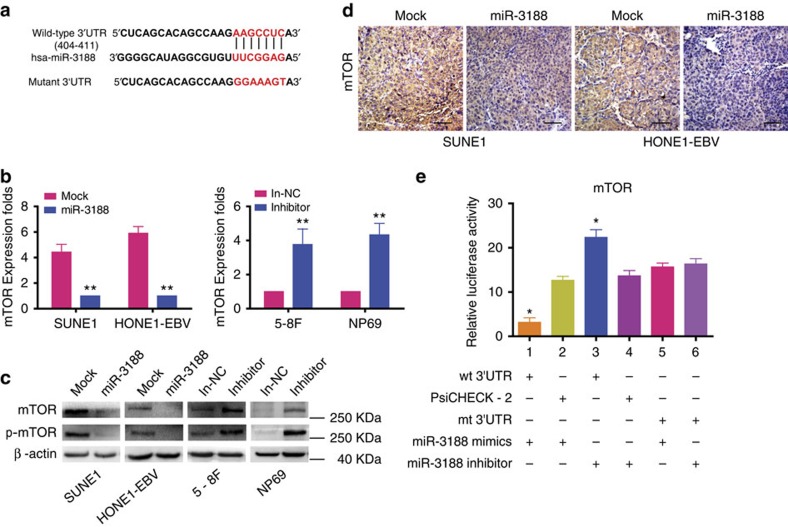
miR-3188 directly regulates mTOR. (**a**) miR-3188 and its putative binding sequences in the 3′UTR of mTOR. A mutation was generated in the complementary site that binds to the seed region of miR-3188. (**b**) mTOR expression was detected by qPCR in miR-3188-overexpressing or miR-3188-inhibited NPC cells, normalized to ARF5. Student's *t*-test. Mean±s.d., **P<0.01. (**c**) Western blots of mTOR and p-mTOR in miR-3188-overexpressing or miR-3188-inhibited NPC and NP69 cells, β-actin was used as a loading control. (**d**) mTOR expression was evaluated by immunohistochemistry in xenografts derived from NPC cells. Scale bar: 30 μm. (**e**) Luciferase reporter assay was used to determine miR-3188 direct targeting the mTOR 3′UTR. One-way ANOVA and Dunnett's multiple comparison test. Mean±s.d., *P<0.05.

Through TargetScan and RNAhybrid algorithms, mTOR was predicted to be a direct target of miR-3188 (Fig. 2a). Overexpression of miR-3188 downregulated mTOR mRNA and protein levels as well as p-mTOR levels in HONE1-EBV and SUNE1 cells. Conversely, miR-3188 downregulation elevated mTOR and p-mTOR levels in 5-8F and NP69 cells (Fig. 2b,c). Consistent with *in vitro* results, immunohistochemistry of xenografts generated from HONE1-EBV-3188 and SUNE1-3188 cells revealed a marked reduction in mTOR expression (Fig. 2d). Simlarly, cotransfection miR-3188 mimics significantly decreased mTOR luciferase reporter activity (Fig. 2e, lanes 1 and 2; One-way ANOVA and Dunnett's multiple comparison test, *P*<0.05), while miR-3188 inhibitor had the opposite effect (Fig. 2e, lanes 3 and 4; One-way ANOVA and Dunnett's multiple comparison test, *P*<0.05). These effects on luciferase activity were abrogated when cotransfected with mutated mTOR reporter (Fig. 2e, lanes 5 and 6, One-way ANOVA and Dunnett's multiple comparison test, *P*=0.078). Collectively, these data suggest that miR-3188 exerts its effects in NPC through direct suppression of mTOR.

### mTOR overexpression reverses the suppression of miR-3188

**Figure 3:**
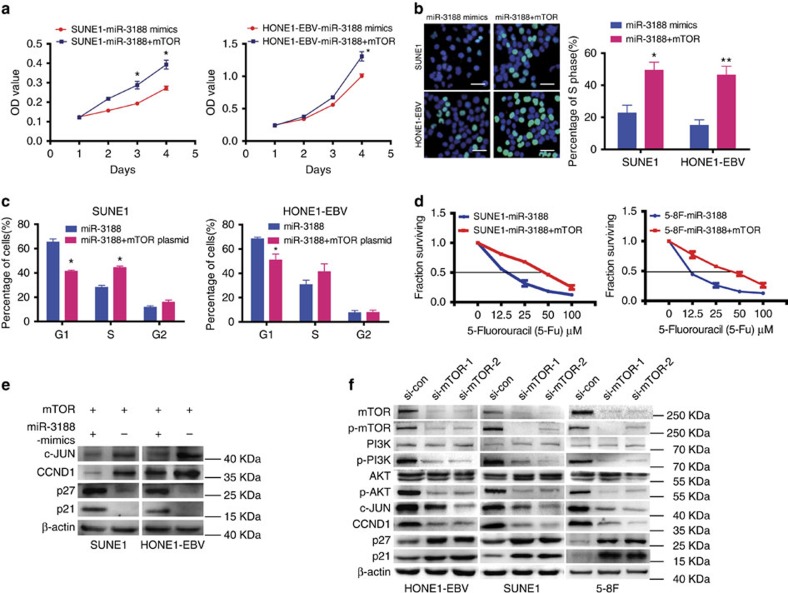
Ectopic expression of mTOR mitigates miR-3188 suppression of NPC proliferation with inverse expression of cell-cycle pathway genes. MTT assays (**a**), EdU incorporation assays (**b**) and FACS assays (**c**) of NPC cells were performed after transfection with NC, ectopic mTOR or miR-3188 as indicated. Scale bar: 15 μm. Parametric generalized linear model with random effects, Student's *t*-test, One-way ANOVA and Dunnett's multiple comparison test. Mean±s.d., *P<0.05; **P<0.01. (**d**) mTOR overexpression reversed miR-3188 NPC cell sensitivity to 5-FU compared to control cells. (**e**) Cell-cycle regulators including c-JUN, CCND1, p27 and p21 were detected by western blot after transfection with NC, ectopic mTOR or miR-3188 as indicated. β-actin served as the internal control. (**f**) Western blot of endogenous mTOR, p-mTOR, PI3K, P-PI3K, AKT, P-AKT, CCND1 and c-JUN protein expression levels in HONE1, SUNE1 and 5-8F cells treated with si-mTOR or si-control. β-actin served as a loading control.

Transiently transfecting mTOR into miR-3188-overexpressing NPC cells (Supplementary Fig. 2A) enhanced cell proliferation by MTT (Fig. 3a) and EdU incorporation assays (Fig. 3b) as well as promoted G1 to S cell-cycle transition (Fig. 3c). mTOR overexpression significantly reversed the 5-FU sensitizing effects of miR-3188 in SUNE1 and 5-8F cells (Fig. 3d). Furthermore, we found that mTOR overexpression induced expression of c-JUN and CCND1 but reduced p27 and p21 (Fig. 3e). These results indicate that mTOR overexpression can overcome NPC cell growth suppression induced by miR-3188.

Subsequently, we found that levels of mTOR, p-mTOR, p-PI3K, p-AKT, CCND1 and c-JUN were significantly decreased, while p27 and p21 were elevated after mTOR siRNA treatment (Fig. 3f). These results were consistent with miR-3188 overexpression, suggesting that mTOR is a direct target of miR-3188 responsible for suppressing cell growth and inducing NPC sensitization to 5-FU.

### c-JUN inhibites miR-3188 by binding to its promoter region

**Figure 4:**
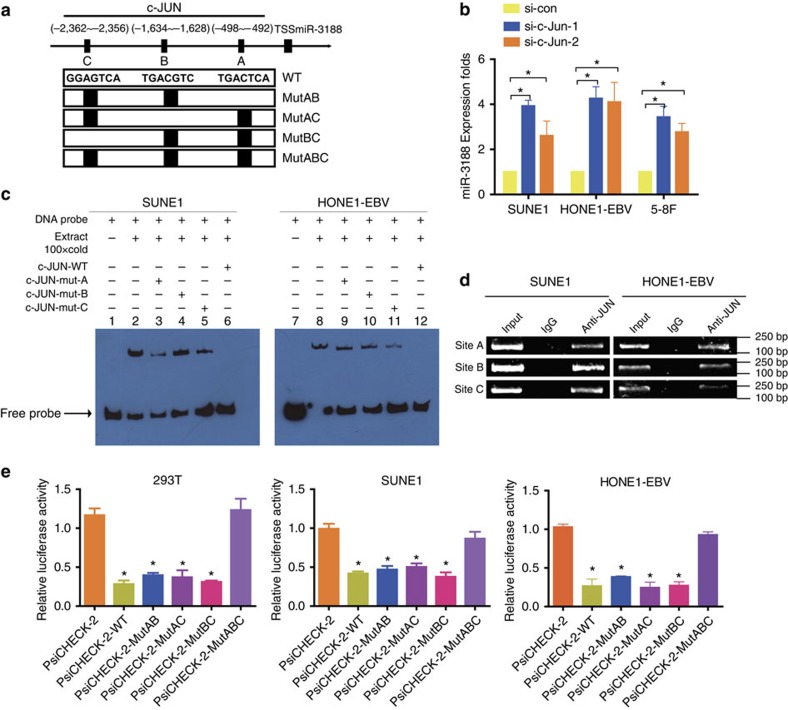
Confirmation of c-JUN transcription factor-binding sites in the miR-3188 promoter region. (**a**) Schematic representation of the promoter regions of miR-3188 with the putative C-JUN TFBSs (**a**,**b** and **c**) and the structure of the wild-type (WT) and TFBS mutant (MutAB, MutAC, MutBC and MutABC) luciferase reporters driven by the promoter. (**b**) Knocking down c-JUN expression by siRNA stimulated miR-3188 expression in SUNE1, HONE1-EBV and 5-8F cells. Student's *t*-test. Mean±s.d., *P<0.05. (**c**) EMSA result was shown from nuclear proteins extracted from SUNE1 and HONE1-EBV cells after incubation with individual DIG-ddUTP-labelled oligonucleotide probes (lanes 2–6, 8–12). The free probe of labelled c-JUN was run in lanes 1 and 7 as a control. A 100-fold excess of unlabelled c-JUN-WT was used to compete with c-JUN binding (lanes 6 and 12, compared with lanes 2 and 8). A 100-fold excess of unlabelled mutated c-JUN-A, c-JUN-B and c-JUN-C was used to compete with binding of respective labelled probes (lanes 3–5 and lanes 9–11 compared with lanes 2 and 8). (**d**) PCR gel showing amplification of c-JUN-binding sites A, B and C after ChIP using antibody against c-JUN. The gel figures were accompanied by the locations of molecular weight markers. (**e**) Relative luciferase activity of the indicated promoter vectors in 293T, HONE1-EBV and SUNE1 cells transfected with c-JUN plasmids. One-way ANOVA and Dunnett's multiple comparison test. Mean±s.d., *P<0.05.

To test the transcriptional regulatory mechanisms of miR-3188 expression, UCSC, PROMO and TFSEARCH bioinformatics software was utilized to analyze a 3-kb region upstream of the transcription start site of miR-3188. Three c-JUN-binding motifs at −492 to −498, −1,628 to −1,634 and −2,356 to −2,362 were identified inside the putative miR-3188 promoter region. These three transcription factor-binding sites (TFBSs) were named A, B and C (Fig. 4a). To examine the role of c-JUN in regulating miR-3188, we first used small-interfering RNAs (siRNAs) to suppress c-JUN expression in HONE1-EBV, SUNE1 and 5-8F cells (Supplementary Fig. 2B). Next, quantitative PCR (qPCR) analysis indicated that miR-3188 expression was markedly increased in all lines after c-JUN knock-down (Fig. 4b), suggesting that c-JUN is an upstream regulator of miR-3188.

To identify whether c-JUN-A, c-JUN-B or c-JUN-C was functional, we first performed electrophoresis mobility shift assay (EMSA) experiment to check whether nuclear extracts of SUNE1 and HONE1-EBV cells could bind to predicted sites A, B or C. As shown in Fig. 4c, a shift band was formed when the probe of digoxygenin (DIG)-ddUTP-labelled c-JUN was incubated with the nuclear protein extracted from SUNE1 and HONE1-EBV cells (lanes 2 and 8), whereas the band was nearly gone when unlabelled oligonucleo tides of c-JUN were added as binding competition (lanes 6 and 12). Bands were not affected when mutated A, B or C was added to compete with DIG-ddUTP-labelled A, B or C in SUNE1 cell and HONE1-EBV cell (lanes 3–5, lanes 9–11). The EMSA results demonstrate that the three predicted c-JUN-binding sites in the promoter region of miR-3188 were functional. Chromatin immunoprecipitation (ChIP) assays further confirmed that c-JUN protein was recruited to all the three binding sites in the putative miR-3188 promoter in SUNE1 and HONE1-EBV (Fig. 4d). Furthermore, a reduction of the wild-type miR-3188 promoter luciferase activity was observed on upregulation of c-JUN in the HEK293T, SUNE1 and HONE1-EBV cell lines (One-way ANOVA and Dunnett's multiple comparison test, *P*<0.05). A similar effect was observed when sites A and B, sites A and C, sites B and C were mutated respectively in 293T, SUNE1 and HONE1-EBV cells (One-way ANOVA and Dunnett's multiple comparison test, *P*<0.05) (Fig. 4e). These data indicate that c-JUN binds to specific promoter TFBS of miR-3188 and inhibits transcription.

Taken together, our data suggest c-JUN is involved in miR-3188 transcription, and all the three c-JUN-binding sites of the miR-3188 promoter are functional in SUNE1 and HONE1-EBV cells.

### FOXO1 acts as a tumour suppressor reducing cell growth

The latent membrane protein 1 (LMP1) of EBV is closely associated with NPC pathogenesis. To determine whether the expression of FOXO1 was influenced by EBV, HONE1 and HONE1-EBV lysates were examined by western blot. No obvious difference in FOXO1 was observed for the EBV infected variant of the line (Supplementary Fig. 3A). In addition, no significant changes in FOXO1 protein were noted after siLMP1 or pSG5-LMP1 transfection in HONE1-EBV cells (Supplementary Fig. 3B). On the basis of these data, we infer that total FOXO1 levels are not modulated by LMP1 in NPC.

**Figure 5:**
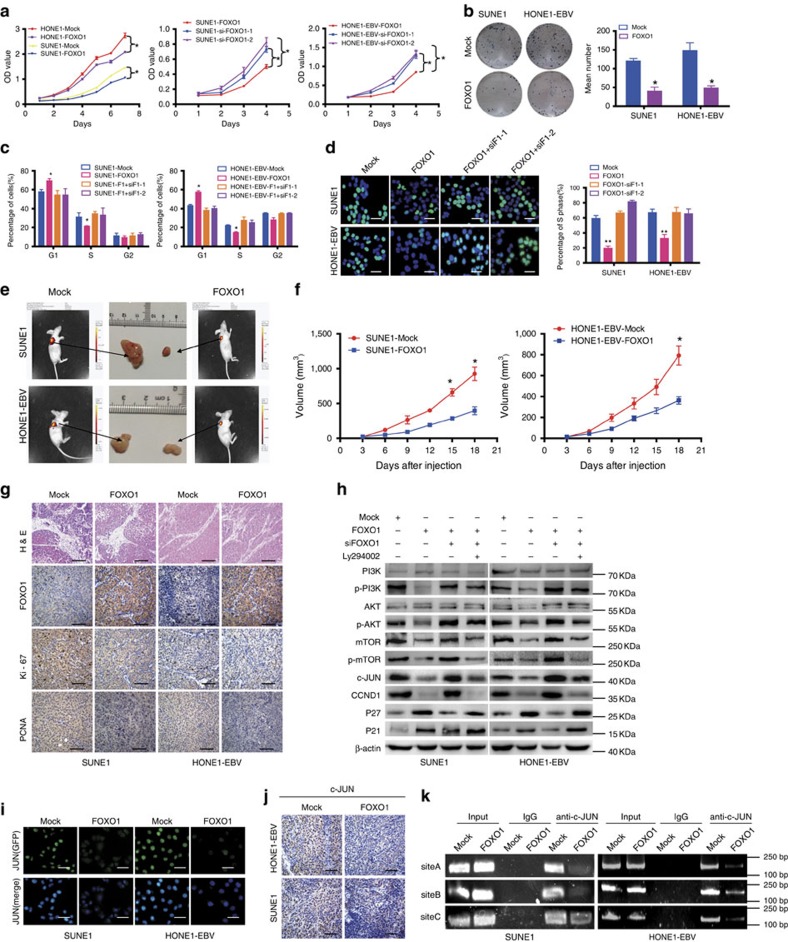
FOXO1 suppresses proliferation of NPC cells by modulating the PI3K/AKT pathway. MTT assays (**a**), colony formation assay (**b**), FACS (**c**) and EdU incorporation assays (**d**) of NPC cells were performed after transfection with mock and/or FOXO1, siRNA against FOXO1 as indicated. Scale bar: 15 μm. Parametric generalized linear model with random effects, Student's *t*-test, One-way ANOVA and Dunnett's multiple comparison test. Mean±s.d., *P<0.05, **P<0.01. (**e**) Tumorigenicity of NPC cells overexpressing FOXO1 was markedly reduced *in vivo;* *n*=5/group. (**f**) Tumour volume was periodically measured for each mouse and growth curves were plotted. Parametric generalized linear model with random effects. Mean±s.d., *P<0.05. (**g**) Representative H&E staining of primary cancer tissues are shown as well as immunohistochemistry (IHC) detection of FOXO1, Ki67 and PCNA. Magnification × 400. Scale bar: 30 μm. (**h**) PI3K, p-PI3K, AKT, p-AKT mTOR, p-mTOR, c-JUN, CCND1, p27 and p21 were measured by western blot after transfection with mock and FOXO1, siFOXO1 or Ly294002 as indicated. β-actin served as a loading control. (**i**) Immunofluorescent images of SUNE1-FOXO1 and HONE1-EBV-FOXO1 and their control cells stained for c-JUN (green) and DAPI (blue). Scale bar: 15 μm. (**j**) Expression of c-JUN was evaluated by immunohistochemistry in tumour tissues derived from NPC mouse models. Magnification × 400. Scale bar: 30 μm. (**k**) ChIP assay of SUNE1 and HONE1-EBV cells treated with FOXO1. c-JUN binding was confirmed by PCR with primers specific for the three sites.

To evaluate its functional significance on cell proliferation, we used a lentiviral vector overexpress FOXO1 in HONE1-EBV, SUNE1 and 5-8F cell lines (Supplementary Fig. 3C). Significant upregulation was confirmed for each line (Supplementary Fig. 3D), which markedly inhibited cell growth and cell-cycle G1/S transition in NPC cells by MTT (Fig. 5a), colony formation (Fig. 5b), flow cytometry (Fig. 5c) and EdU incorporation assays (Fig. 5d). Further, we used siRNA to knock-down FOXO1 (Supplementary Fig. 3E) and found siFOXO1s could reverse the cell-growth suppresion mediated by ectopic expression (Fig. 5a,c,d).

To further confirm the growth-suppressive effect of FOXO1, we performed *in vivo* tumorigenesis experiment in nude mice. Tumour volumes and growth rates were significantly decreased in tumours derived from FOXO1-overexpressing HONE1-EBV and SUNE1 cells (Fig. 5e,f and Supplementary Fig. 3F). These tumours also exhibited a reduction in Ki67 and PCNA expression by immunohistochemistry (Fig. 5g). These results suggest that FOXO1 exerts a significant inhibitory effect on tumorigenesis *in vivo*.

FOXO1 has been reported to induce the PI3K/AKT signalling in gastric cancer^21^, thus we sought to examine this effect in NPC. Overexpression of FOXO1 significantly reduced the levels of p-PI3K, p-AKT, mTOR and p-mTOR. Furthermore, we found that ectopic FOXO1-reduced expression of c-JUN and CCND1 but upregulated p21 and p27. Interestingly, the opposite results were observed after siRNA-mediated suppression of ectopic FOXO1 (Fig. 5h). Further, the specific PI3K inhibitor Ly294002 significantly reversed the expression of p-PI3K, p-AKT, mTOR, p-mTOR, c-JUN, CCND1, p21 and p27 (Fig. 5h).

As a downstream regulator of the PI3K/AKT pathway, c-JUN has been observed to directly suppress miR-3188 expression in NPC. We used immunofluorescence to confirm reduced expression of c-JUN in FOXO1-overexpressing NPC cells (Fig. 5i). This was also confirmed by immunohistochemistry of FOXO1-overexpressing tumour tissues derived from NPC mouse models (Fig. 5j). Finally, ChIP assay revealed less c-JUN binding to the miR-3188 promoter in FOXO1-overexpressing NPC cells compared to control cells (Fig. 5k).

All the results suggest that FOXO1 regulates NPC cell proliferation and cell-cycle progression through the PI3K/AKT/c-JUN pathway.

### miR-3188 is induced by FOXO1 through PI3K/AKT/c-JUN

**Figure 6:**
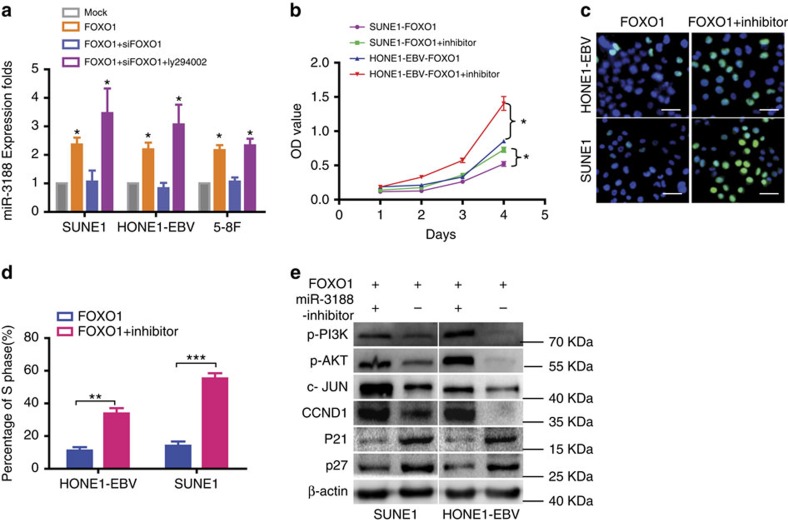
FOXO1 modulates miR-3188 expression in NPC. (**a**) Levels of miR-3188 were dected by qPCR in FOXO1, siFOXO1 or Ly294002 treated NPC cells. One-way ANOVA and Dunnett's multiple comparison test. Mean±s.d., *P<0.05. MTT assays (**b**) and EdU incorporation assays (**c**,**d**) of NPC cells were performed after transfection with FOXO1 or both FOXO1 and inhibitor. Scale bar: 15 μm. Parametric generalized linear model with random effects, Student's *t*-test, mean±s.d., *P<0.05; **P<0.01; ***P<0.001. (**e**) Expression of p-PI3K, p-AKT, c-JUN and CCND1 were increased, but p21 and p27 were remarkably reduced following FOXO1 overexpression and miR-3188 inhibition. β-actin served as a loading control.

To investigate the effect of FOXO1 on miRNAs in NPC, we used miRNA chip on SUNE1 cells after FOXO1 overexpression. This analysis identified eighty-two miRNAs, which were markedly dysregulated in FOXO1-overexpressing cells, including miR-3188, miR-29c, miR-141 and miR-200c (Supplementary Fig. 4). miR-3188 was confirmed as a positive modulator of FOXO1 via qRT-PCR in NPC cells treated with Mock, FOXO1 or both FOXO1 and siFOXO1 (Fig. 6a). Reduction of miR-3188 by its specific inhibitor could reverse the growth-suppressive effect after ectopic FOXO1 expression in MTT (Fig. 6b) and Edu assays (Fig. 6c,d). Western blot analysis showed that treatment with a miR-3188 inhibitor increased expression of p-PI3K, p-AKT, c-JUN and CCND1, but reduced p27 and p21 levels in FOXO1-overexpressing NPC cells (Fig. 6e). These results indicate that miR-3188 is induced by FOXO1 and suppresses NPC cell growth.

Specific PI3K inhibitor Ly294002 reversed the changes in miR-3188 expression in NPC cells with both FOXO1 overexpression or silencing (Fig. 6a). This suggests that FOXO1 positively regulated the expression of miR-3188 through the PI3K/AKT pathway.

Taken together, these results support that miR-3188 expression is induced by FOXO1 through PI3K/AKT/c-JUN signalling.

### Pathoclinical features of miR-3188 expression

**Figure 7:**
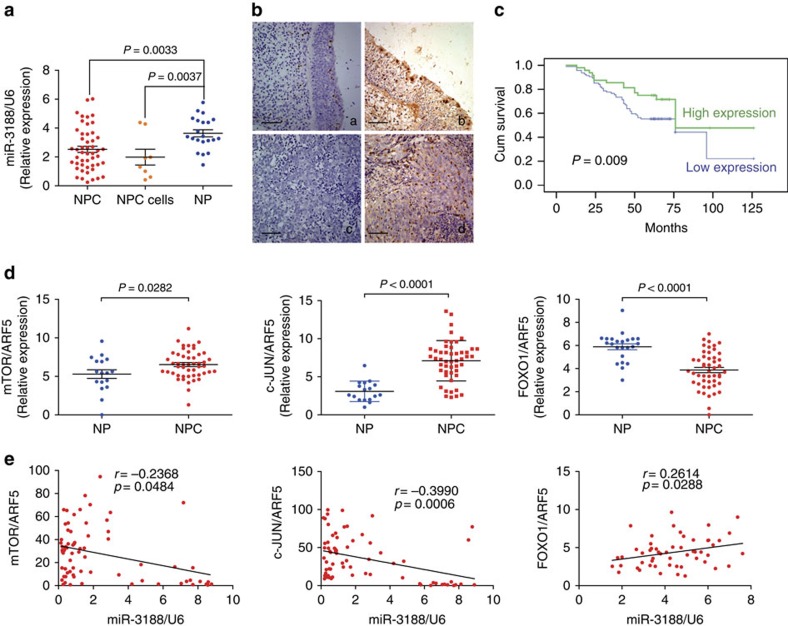
Pathoclinical features of miR-3188 expression and its correlation with other key genes. (**a**) Compared with NP tissues, miR-3188 expression was markedly decreased in NPC tissues and NPC cells. Student's *t*-test, mean±s.d., *P<0.05; **P<0.01. (**b**) miR-3188 expression in NPC and NP samples. (a) Weak expression of miR-3188 in NP samples; (b) strong staining of miR-3188 in NP samples; (c) negative expression of miR-3188 in NPC samples; (d) strong staining of miR-3188 in NPC samples (original magnification × 400). Scale bar: 30 μm. (**c**) Kaplan–Meier survival analysis of overall survival of 142 NPC patients on the basis of miR-3188 expression. Log-rank test was used to calculate *P* values. (**d**) Levels of mTOR, c-JUN and FOXO1 were detected by qPCR in NPC and NP tissue specimens, normalized to the U6 and ARF5, respectively. Student's *t*-test, mean±s.d., *P*=0.0282; *P*=0.0001; *P*=0.0001. (**e**) Correlations between miR-3188, mTOR, c-JUN and FOXO1 expression levels were calculated. Two tailed Spearman's correlation analysis. Mean±s.d., *P*=0.0484; *P*=0.0006; *P*=0.0288.

Levels of miR-3188 were significantly decreased in 8 NPC cell lines and NPCs compared to NP tissues by qPCR analysis (Student's *t*-test, *P*=0.00037, *P*=0.00033, respectively) (Fig. 7a). Further, *in situ* hybridization assay confirmed reduced expression of miR-3188 in NPC tissues compared to NP tissues (Fig. 7b; Table 1). Clinical characteristics of the NPC patients are summarized in Table 2. We did not find a significant association between miR-3188 expression level and patient age, sex, clinical stage (I–II versus III–IV), lymph node metastasis (N classification; N0–N1 versus N2–N3) or distant metastasis stage (M classification) in the 142 NPC cases. However, we observed that reduced-miR-3188 expression was negatively correlated with tumour size (T classification; χ^2^ test, *P*=0.011) (Table 2). Subsequently, we found that NPC patients with high miR-3188 expression had longer survival times than those of patients with low miR-3188 levels (Log-rank test, *P*=0.009, Fig. 7c).

### Correlation of miR-3188 with other key genes

To further confirm the relationship between miR-3188, mTOR, FOXO1 and c-JUN, we analyzed their mRNA expression in NPC and NP samples. As shown in Fig. 7d, mTOR and c-JUN expression were significantly higher in NPC than in NP samples (Student's *t*-test, *P*=0.0282, *P*<0.0001, respectively), while FOXO1 expression was significantly lower in NPC samples (Student's *t*-test, *P*<0.0001). miR-3188 expression was positively correlated with FOXO1 expression (Fig. 7e; Spearman's correlation coefficient, *P*=0.0326), but negatively associated with mTOR (Fig. 7e; Spearman's correlation coefficient, *P*=0.0288) and c-JUN expression (Fig. 7e; Spearman's correlation coefficient, *P*=0.0006) in the same NPC specimens.

## Disscussion

miRNAs have been linked with various types of cancer^22^,^23^. However, the roles and molecular mechanisms of newly identified miR-3188 in carcinogenesis have not been reported. In this study, we found that miR-3188 not only significantly inhibited proliferation and G1/S cell-cycle transition of NPC cells *in vitro* but also suppressed tumourigenicity *in vivo*. Furthermore, we also found that miR-3188 overexpression sensitized NPC cells to 5-FU, but not DDP. These results suggest that miR-3188 functions as a potential tumour suppressor in NPC.

It is well established that cell-cycle progression is a predominant factor promoting tumour cell proliferation and inducing chemotherapeutic resistance to 5-FU and DDP^24^. The biological functions of miR-3188 identified in this study provide a mechanism for its role in carcinogenesis. miR-3188 forms a negative feedback loop via key oncogenic genes and signal including mTOR, PI3K/AKT and c-JUN, which suppress cell-cycle signal transition, thus inhibiting cell growth and sensitizing cells to 5-FU. However, miR-3188 overexpression did not affect NPC cells response to DDP, which might attributed to the fact that miR-3188 induces ZEB2 expression and thus promotes an epithelial–mesenchymal transition (EMT)-like process in NPC (Supplementary Fig. 5). While EMT has been widely studied for its role in early development and cancer metastasis, it can also affect resistance to platinum-based therapies^25^. The development of DDP resistance in NPC cells is accompanied by inducible EMT-like changes with an increased metastatic potential *in vitro*^26^.

mTOR functions as an oncogene in many tumour types^27^,^28^,^29^, including NPC^30^. The PI3K/Akt signalling pathway controls fundamental cellular processes, such as cell survival, growth, proliferation, cell repair, cell migration and angiogenesis, and is constitutively activated in nearly all cancer types^31^,^32^,^33^. Activation of PI3K/Akt/mTOR signalling through mutation of pathway components as well as through activation of upstream signalling molecules occurs in a majority of cancers^34^,^35^. The existence of a negative feedback loop between mTOR and PI3K/AKT has been demonstrated in many reports^36^,^37^,^38^. In our investigation, we found that miR-3188 directly targets mTOR and suppresses PI3K/AKT signalling. This finding contrasted previous studies in breast cancer that mTOR suppresses PI3K/AKT signalling^36^,^37^. Expression of PI3K/AKT downstream components including cell-cycle factors, c-JUN and p-mTOR were dysregulated in mTOR-silenced NPC cells, a pattern consistent with miR-3188 overexpression. Furthermore, overexpression of mTOR reversed the inhibitory growth effect mediated by miR-3188 and promoted NPC cell proliferation. These results support that miR-3188 directly targets mTOR to suppress PI3K/AKT signalling, especially downstream cell-cycle factors c-JUN and p-mTOR.

An analysis of a region upstream to the miR-3188 locus revealed multiple putative binding sites for c-JUN, an essential regulator of cell proliferation^39^, invasiveness, metastasis^40^ and PI3K/AKT signalling^11^,^41^. Subsequent experiments confirmed that c-JUN could negatively regulate miR-3188 expression by directly binding its promoter region. These results thus indicate that miR-3188 can induce its own expression though a complex miR-3188-mTOR–pPI3K/AKT-c-JUN loop in NPC pathogenesis.

Although the tumour suppressive activity of FOXO1 has been well characterized for some cancers, little is known of its function and molecular mechanisms in NPC. Similar to previous reports^17^,^18^, FOXO1 significantly inhibited NPC cell G1/S cell-cycle transition and proliferation *in vitro* and *in vivo*. Upstream cell-cycle signalling from the PI3K/AKT pathway was decreased in NPC cells after FOXO1 overexpression. These results are in contrast to a previous report in gastric cancer^21^. Two downstream regulators of PI3K/AKT signalling, c-JUN and p-mTOR, as well as total mTOR levels were reduced in NPC. Taken together, FOXO1 suppresses NPC cell growth by blocking PI3K/AKT-mediated cell-cycle transition, a role consistent with that of miR-3188. To our knowledge, the detailed mechanisms for FOXO1-induced inhibition of mTOR protein have not been previously documented.

To investigate the effect of FOXO1 on miRNAs, we used a miRNA chip after FOXO1 overexpression in SUNE1 NPC cells. Among miRNAs differentialy express were miR-141 (ref. 42) and miR-29c (ref. 43), which have been reported to be involved in NPC carcinogenesis. We observed a marked upregulation of miR-3188 in FOXO1-overexpressing NPC cells and further confirmed these results by qPCR. We observed that miR-3188 could not induce the expression of FOXO1, which suggested FOXO1 could be an upstream regulator of miR-3188. Suppression of miR-3188 partially rescued the inhibitory growth effects of FOXO1 and promoted cell proliferation. This was achieved by suppressing mTOR-mediated activation of p-PI3K/AKT/p-mTOR, c-JUN and cell-cycle signalling. These data demonstrated that miR-3188 is a downstream effector of FOXO1 signalling and participates in FOXO1-induced growth suppression in NPC.

We observed that miR-3188 was negatively modulated by c-JUN, a downstream regulator of the PI3K/AKT pathway. Interestingly, this signalling cascade is suppressed by FOXO1. We suspected that miR-3188 is induced by FOXO1 by inhibiting PI3K/AKT/c-JUN signalling. Indeed, we found that miR-3188 expression was significantly reduced after PI3K inhibition in FOXO1-overexpressing NPC cells.

Consistent with their roles *in vitro* and *in vivo*, we observed that miR-3188 levels were significantly decreased in NPC tissues compared to NP tissues. Furthermore, reduced miR-3188 expression was negatively correlated with T classification and positively associated with the survival time of NPC patients. Patients that exhibited low miR-3188 expression had an overall shorter survival time compared to patients with high miR-3188 expression. Further, we also observed reduced FOXO1 but elevated mTOR and c-JUN levels in clinical NPC tissues compared to NP samples. miR-3188 was positively correlated with FOXO1 expression but negatively correlated with mTOR and c-JUN mRNA levels. Altogether these results suggest that miR-3188 exerts an important role in NPC tumorigenesis between FOXO1, c-JUN and mTOR dysfunction.

**Figure 8:**
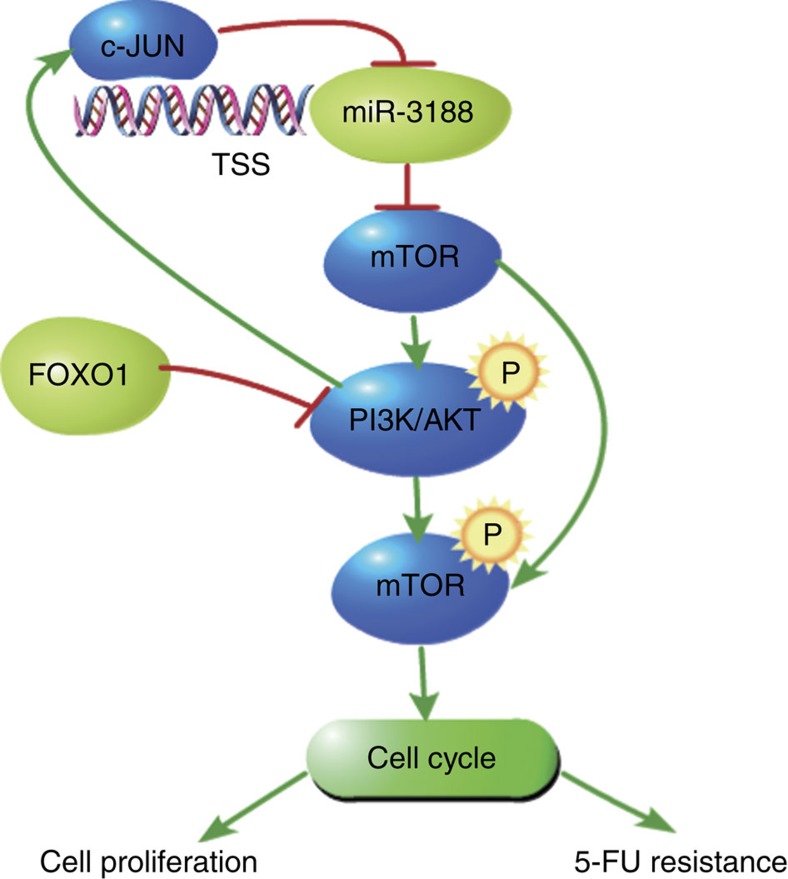
A schematic for an atypical miR-3188-mTOR–p-PI3K/AKT-c-Jun feedback loop mediated by FOXO1. miR-3188 downregulates p-PI3K/AKT signalling by directly targeting its upstream regulator mTOR. PI3K/AKT signalling in turn downregulates miR-3188 by c-JUN directly binding to its promoter. This feedback loop also mediates by FOXO1 suppression.

As summarized in our working model in Fig. 8, miR-3188 does not suppress cell growth and enhance chemotherapeutic sensitization to 5-FU alone. Instead, miR-3188 forms a negative feedback loop through mTOR/PI3K/AKT/c-JUN signalling that is modulated by FOXO1. There is an increasing appreciation that miRNAs form regulatory motifs with protein regulators, which underlie pathogenesis when dysregulated^44^. Therefore, context is important for understanding the role of specific miRNAs in regulatory networks. We hypothesize that once induced by FOXO1, the miR-3188-mediated feedback loop allows NPC cells to become less autonomous reducing cell proliferation.

## Methods

### Cell culture and synchronization

Eight EBV-negative NPC cell lines (5-8F, 6-10B, CNE1, CNE2, C666-1, HONE1, HNE1 and SUNE1) and HEK293T cells were obtained from the Cancer Research Institute of Southern Medical University, Guangzhou, China. NPC cell lines were cultured in RPMI-1640 (Invitrogen) supplemented with 10% foetal calf serum (FCS; Hyclone, Invitrogen), while HEK293T cells were grown in Dulbecco's modified Eagle's medium (Invitrogen) with 10% newborn calf serum (NCS). NP69, an immortalized human nasopharyngeaepithelial cell line, was cultured in defined KSFM medium supplemented with epidermal growth factor (Invitrogen, Carlsbad, USA). The immortalized human nasopharyngeaepithelial cell line CXCW-1489 was purchased in SiXin, Shanghai, China and cultured in 10% FCS (Hyclone, Invitrogen). EBV-positive NPC cell line HONE1-EBV was kindly provided by Professor S.-W. Tsao, University of Hong Kong. All cell lines were incubated in a humidified chamber with 5% CO_2_ at 37 °C.

For synchronizing the cells into G0 phase, 10% FCS RPMI-1640 was replaced with 0.1% FCS RPMI-1640 24–48 h after indicated transfection. Cells were serum starved for 48 h. Samples for western blotting and flow cytometry were collected at indicated time points after cell-cycle release.

### Tissue specimens

Forty nine (49) primary fresh NPC samples with tumour node metastasis (TNM) staging and 20 non-cancerous fresh nasopharyngeal samples were collected from the People's Hospital of Zhongshan City, China, at the time of diagnosis before any therapy. All fresh samples were immediately preserved in liquid nitrogen. One hundred and forty two (142) paraffin-embedded NPC specimens and 36 paraffin-embedded were obtained from the People's Hospital of Zhongshan City, Guangdong Province, China. In the 142 NPC cases, there were 99 male and 43 female with age ranging from 20 to 84 years (median, 58.9 years). For the use of these clinical materials for research purposes, prior consent from the patients and approval from the Ethics Committee of the People's Hospital of Zhongshan City were obtained. All specimens had confirmed pathological diagnosis and were staged according to the 1997 NPC staging system of the UICC.

### *In situ* hybridization

The expression levels of miR-3188 in 142 paraffin-embedded NPC specimens and 36 paraffin-embedded NP tissues were detected by *in situ* hybridization. Tissue sections were dewaxed in xylene, rehydrated through an ethanol gradient and then treated with 3% H_2_O_2_ for 10 min. Subsequently, sections were treated with pepsin dilution in 3% fresh citrate buffer at 37 °C for 30 min and then washed. Further, hybridization with DIG-labelled miRCURY LNA probes (probe sense: Bis-P22401; Biosense Bioscience Co. Ltd, Guangzhou, China) was performed overnight at 37 °C after pre-hybridization was carried out using 20 μl of pre-hybridization solution for 2 h at 37 °C. Sections were subjected to high stringency washes with 2 × SSC, 0.5 × SSC and 0.2 × SSC for 5, 15 and 15 min at 37 °C. Afterwards, the sections were incubated in blocking solution for 30 min at 37 °C and then incubated with alkaline phosphatase-conjugated sheep anti-DIG Fab fragments for 60 min at room temperature (RT). Positive staining of miR-3188 was observed by adding BM purple AP substrate (Roche, Basel, Switzerland) according to the manufacturer's instructions.

### Lentivirus production and infection

Lentiviral particles carrying hsa-miR-3188 precursor and pGC-FU-FOXO1-RFP vector and their flanking control sequence (Mock for short) were constructed by GeneChem (Shanghai, China).

SUNE1, HONE1, HONE1-EBV and 5-8F cells were infected with lentiviral vector, and polyclonal cells with green or red fluorescent protein signals were selected for further experiments using fluorescence-activated cell sorting. Hsa-miR-3188 expression was confirmed by qPCR and the levels of FOXO1 (Cat. No. 2880, 1:1,000, CST) protein were measured using western blotting.

### Cell transfection

siRNA for FOXO1, c-JUN and mTOR or miR-3188 mimics and its inhibitor were designed and synthesized by RiboBio Inc. (Guangzhou, China) (Supplementary Table 1). mTOR and c-JUN plasmids were purchased in Biosense Technologies (Guangzhou, China). LMP1 plasmid was kindly provided by Prof. Yongguang Tao, Cancer Institute of Xiangya Medical School, Central Southern University. PI3K inhibitor Ly294002 was purchased from Sigma. Twenty-four hours before transfection, NPC cells were plated onto a 6- or 96-well plate (Nest, Biotech, China) at 30–50% confluence. siRNA, mTOR plasmid, c-JUN plasmid, or miRNAs were then transfected at a working concentrations of 100 nM using TurboFect siRNA Transfection Reagent (Fermentas, Vilnius, Lithuania) according to the manufacturer's protocol. Cells were collected after 48–72 h for further experiments.

### qRT-PCR

RNA was extracted from the NPC cell lines, tissues and normal nasopharynx tissues by Trizol (Takara Bio, Inc., Shiga, Japan). U6 and ARF5 genes were used as miRNA and gene internal controls, respectively. Cycling conditions were 95 °C for 10 min to activate DNA polymerase, followed by 45 cycles of 95 °C for 15 s, 60 °C (for miR-3188, c-JUN, LMP1 and FOXO1 ) for 15 s and 72 °C for 10 s. Specificity of amplification products was confirmed by melting curve analysis. Independent experiments were done in triplicate. Specific sense primers for miR-3188, c-JUN, LMP1, FOXO1, U6 and ARF5 are shown in Supplementary Table 2.

### Western blotting

Western blot was performed according to a previous description^11^. Antibodies included anti-FOXO1, mTOR, p-mTOR, CCND1, p21, c-JUN, AKT, pAKT (Ser473), PI3K, pPI3K (Tyr458), p27, LMP1, Snail, E-cadherin, N-cadherin ZEB2 and β-actin. The antibodies were listed in Supplementary Table 3. The images were captured with ChemiDocTM CRS+ Molecular Imager (Bio-Rad). All blots in figures were accompanied by the locations of molecular weight/size markers. Original blotting images for the key components of the PI3K/AKT pathway were shown in Supplementary Figs 6–10.

### Immunofluorescent staining

NPC cells grown on coverslips were rinsed with phosphate-buffered saline (PBS) and fixed with cold 4% paraformaldehyde for 5 min at RT. Subsequently, the cells were blocked with Triton X-100 at a concentration of 0.3% for 30 min and incubated with primary monoclonal antibodies c-JUN (Cat. No. 9165, 1:50, CST) in PBS for 2 hrs at RT. After three washes in PBS, the coverslips were incubated for 1 h in the dark room with Alexa Fluor 488 goat anti-rabbit IgG (1:500, Bioworld Technology, Inc.). Further, the coverslips were washed three times and then stained with 4-6-diamidino-2-phenylindole (DAPI) for 5 min at 4 °C. Finally, ECLIP SE 80i fluorescent microscope (Nikon, Japan) was used to observe the expression of c-JUN in NPC cells.

### Cell proliferation and colony formation assays

The MTT assay was used to examine cell viability. NPC cells (1,000/well) were seeded in 96-well plates. For lentivirus-mediated FOXO1 overexpression, the cells were incubated for 1, 2, 3, 4, 5, 6 or 7 days. For transient transfection with siFOXO1, miR-3188 mimics, miR-3188 inhibitor or mTOR plasmid *et al*, the cells were cultured for 1, 2, 3 or 4 days. Subsequently, 20 μl of MTT (5 mg ml^–1^ in PBS) (Sigma, St Louis, MO) solution was added to each well and incubated for 4 h. The formazan crystals formed by viable cells were solubilized in 150 μl dimethyl sulfoxide (Sigma, St Louis, MO) and then the absorbance value (OD) was measured at 490 nm. For colony formation assay, NPC cells were seeded in 6-well culture plates at a density of 100 cells/well and each group had 2 wells. After incubation for 14 days at 37 °C, colonies were washed twice with PBS and stained with hematoxylin solution. The colonies composed of more than 50 cells in a well were counted under a microscope. All the experiments were repeated for at least three times.

### Cell-cycle analysis and EdU incorporation assay

Cell-cycle analyses and EdU incorporation assays were performed according to a previous description^45^. For cell-cycle analysis, a total number of 5 × 10^6^ NPC cells were harvested after 48 h incubation and then washed with cold PBS. The cells were further fixed with 70% ice-cold ethanol at 4 °C overnight. After incubation with PBS containing 10 mg ml^−1^ propidium iodide and 0.5 mg ml^−1^ RNase A for 15 min at 37 °C, fixed cells were washed with cold PBS three times. FACS caliber flow cytometry (BD Biosciences) was used to gain the DNA content of labelled cells. For EdU incorporation assay, proliferating NPC cells were examined using the Cell-Light EdU Apollo 488 or 567 *In Vitro* Imaging Kit (RiboBio) according to the manufacturer's protocol. Briefly, after incubation with 10 μM EdU for 2 h, NPC cells were fixed with 4% paraformaldehyde, permeabilized in 0.3% Triton X-100 and stained with Apollo fluorescent dyes. A total of 5 μg ml^−1^ of DAPI were used to stain cell nuclei for 10 min. The number of EdU-positive cells was counted under a fluorescent microscope in five random fields. All assays were independently performed for three times.

### *In vivo* tumorigenesis in nude mice

A total of 5 × 10^6^ logarithmically growing NPC cells transfected with miR-3188 and FOXO1 or the control (*N*=5 per group) in 0.1 ml 1,640 medium without FBS were subcutaneously injected into the left–right symmetric flank of the mice (BALB/C, nu/nu, 4–5–weeks-old, female). The mice were maintained in a barrier facility on HEPA-filtered racks. The animals were fed an autoclaved laboratory rodent diet. All animal studies were conducted in accordance with the principles and procedures outlined in the Southern Medical University Guide for the Care and Use of Animals under assurance number SCXK (Guangdong) 2008-0002. After 19 days, the mice were killed and tumour tissues were excised and weighed.

### Immunohistochemical staining

Paraffin sections prepared from *in vivo* experiments were used for immunohistochemistry assays to detect protein expression levels of FOXO1, mTOR, Ki67, PCNA or c-JUN proteins. The indirect streptavidin-peroxidase method was used according to the manufacturer's introduction. Immunohistochemically stained tissue sections were examined separately by two pathologists. The antibodies used were rabbit anti-FOXO1 (Cat. No. 1:250, Abcam), anti-mTOR (Cat. No. 04-385, 1:250, millipore), anti-PCNA (Cat. No. 10205-2-AP, 1:30, PTG), anti-Ki67 (Cat. No. Ab16667, 1:100, Abcam) and anti-JUN (Cat. No. 24909-1-AP, 1:250, PTG).

### miRNA array following overexpressed FOXO1

miRNA array was carried out by Gene Co., Ltd (Shanghai, China). Affymetrix Gene Chip Micro 2.0 Array (Affymetrix, Inc., Santa Clara, CA, USA), which provides for 100% miRBase v17 coverage (www.mirbase.org) by a one-color approach, was employed for universal miRNA coverage^46^. Total RNA (6–7 mg) was isolated from FOXO1 and control SUNE1 cells. Statistical analysis was carried out using the open source R-software (http://www.r-project.org) as previous studies have described^47^. The raw data were deposited in GEO database (Accession Number: GSE78742).

### Luciferase reporter assay

mTOR was predicted to be directly regulated by miR-3188 using TargetScan software. A 601-bp fragment of mTOR 3′UTR amplified by PCR primers (Supplementary Table 2) was cloned into psiCHECK-2 vectors (named wt). Site-directed mutagenesis of the miR-3188 binding site in the mTOR 3′UTR (named mt) was performed using GeneTailor Site-Directed Mutagenesis System (Invitrogen). For reporter assays, wt or mt vector and the control vector psiCHECK-2 vector were cotransfected into SUNE1 cells with miR-3188 mimics or inhibitor. Luciferase activity was measured at 48 h after transfection using the Dual-Luciferase Reporter Assay System (Promega Corporation, Madison, WI, USA).

To generate a miR-3188 promoter vector, a 2,472-bp fragment containing the three binding sites of c-JUN was PCR-amplified and inserted into a psiCHECK-2 luciferase reporter vector. In addition, c-JUN-binding site mutation vectors were constructed. These psiCHECK-2-derived vector and c-JUN-expressing vectors were cotransfected into 293T or HONE1-EBV and SUNE1 cells using Lipofectamine 2,000 Reagent (Invitrogen). Primer sequences used for PCR amplification of plasmid construction are listed in Supplementary Table 2.

### Chromatin immunoprecipitation assay

According to the manufacturer's instructions, ChIP assay was performed to examine whether c-JUN combined to miR-3188 promoter by a ChIP assay kit (Millipore, catalog: 17-371). SUNE1 and HONE1-EBV cells with or without lentiviral-mediated ectopic FOXO1 expression were firstly fixed with 1% formaldehyde to covalently crosslink proteins to DNA and then chromatin was harvested from the NPC cells. Crosslinked DNA was sheared to 200–1,000 base pairs in length with sonication and then subjected to an immmmunoselection process, which required the use of Anti-c-JUN antibody (Cat. No. 9165, 1:50, CST). Finally, PCR was used to measure enrichment of DNA fragments in the putative c-JUN-binding sites in the miR-3188 promoter on the basis of the specific primers (Supplementary Table 2).

### EMSA analysis

Binding activity on the promoter region of miR-3188 (c-JUN-A, c-JUN-B and c-JUN-C) was detected by the EMSA Kit (Roche, Switzerland) according to the manufacturer's protocol. Probes used in this study are shown in Supplementary Table 2. The preformed c-JUN recognized probe (Biosense Bioscience Co., Ltd Guangzhou, China) (sense: 5′- GAGCGGATAACAATTTCACACAGG -3′; antisense: 3′- AACACAGCACCTCTTTTTGT -5′) was used as the positive control. Samples without nucleoprotein were used as negative controls. For competition experiments, 100-fold specific oligonucleotide competitor (unlabelled wild-type or mutant c-JUN probes) was added to the binding mixture 10 min before the addition of the labelled probe. Visualized bands were analyzed using a BioSens Gel Imaging System (BIOTOP, China). EMSA analysis was performed at Biosense Bioscience Co. Ltd (Guangzhou, China).

### MTT cytotoxicity assay

*Cis*-diamminedichloroplatinum (circulating, DDP) (Qilu Pharmo Co. Ltd, China) was resuspended in PBS (0.5 mg ml^–1^) and stored at –20 °C. 5-FU (Shanghai Xudong Haipu Pharmaceutical Co. Ltd, China) was added in solution (250 mg ml^–1^) and stored at 4 °C.

Drug sensitivity test was determined by MTT assay. Cells were seeded in 96-well plates in 100 μl RPMI-1640 medium supplemented with 10% FBS at 5 × 10^3^ cells/well. Once cells attached, they were treated with 2.5, 5, 10, 20 or 40 μM Cisplatin (0.5 mg ml^−1^) or 12.5, 25, 50 or 100 μM 5-FU (250 mg ml^−1^) and incubated at 37 °C in 5% CO_2_ for 48 h. Subsequently, 10 μl of MTT (5 mg ml^−1^) (Sigma, St Louis, MO, USA) was added to each well, and the plates were incubated at 37 °C for 4 h. At the end of incubation, supernatants were removed and 100 μl of dimethyl sulfoxide (Sigma) was added to each well. The absorbance value (OD) of each well was measured at 490 nm. The calculated rates were then used for curve fitting and half maximal inhibitory concentration (IC50) calculations. Experiments were carried out three times.

### Treatment experiments on nude mice

*In vivo* experiments were approved by the Animal Care and Use Committee of Southern Medical University. All mice (BALB/C, nu/nu) were 5-weeks-old, female, 14–16 g in bodyweight and provided by the Central Animal Facility of Southern Medical University. To establish an NPC mouse model, 6 × 10^5^ miR-3188-overexpressing SUNE1 cells or their controls were intraperitoneal injected in 0.2 ml buffered saline into the mice (*N*=18 each). Tumours were allowed to grow for 3 days and then the animals were divided into four groups (Control cells group (Mock)+Normal saline (NS), miR-3188+NS, Mock+5-FU and miR-3188+5-FU; *N*=9/group) for therapy testing. Mice were intraperitoneal injected with NS or 5-FU every 3 days, respectively. Changes in mouse bodyweight and survival time were measured.

### Statistical analysis

Statistical analyses were performed with the SPSS 13.0 statistical software package (SPSS Inc. Chicago, IL, USA). Data are expressed as the mean±s.d. from at least three independent experiments. Comparisons between two groups were performed using Student's *t*-test, one-way ANOVA (analysis of variance) analysis for multiple group and parametric generalized linear model with random effects for tumour growth and MTT assay. Associations between miR-3188 and mTOR or miR-3188 and FOXO1 gene or miR-3188 and c-JUN were analyzed using Spearman's correlation coefficient. Survival analysis was performed using the Kaplan–Meier method. All statistical tests were two-sided, and single, double and triple asterisks indicate statistical significance—*P<0.05, **P<0.01 and ***P<0.001.

## Additional Information

**How to cite this article:** Zhao, M. *et al*. miR-3188 regulates nasopharyngeal carcinoma proliferation and chemosensitivity through a FOXO1-modulated positive feedback loop with mTOR–p-PI3K/AKT-c-JUN. *Nat. Commun.* 7:11309 doi: 10.1038/ncomms11309 (2016).

## Supplementary Material

Supplementary Information Supplementary Figures 1-10 and Supplementary Tables 1-3

## Figures and Tables

**Table 1:** The expression of miR-3188 in NPC compared to NP tissues.

**Group**	**Cases (*****n)***	**miR-3188 expression**		P* **value**
		**Low expression**	**High expression**	
Cancer	142	94 (48.6%)	48 (51.4%)	0.000
Normal epithelium	36	11 (87.1%)	25 (12.9%)	

NPC, Nasopharyngeal carcinoma; NP, normal epithelium.

^*^χ^2^ test was applied to access the expression of miR-3188 in NPC and NP.

**Table 2:** Correlation between the clinicopathologic characteristics and expression of miR-3188 in NPC.

**Characteristics**	***n***	**miR-3188 expression**		P*value
		**High**	**Low**	
*Age (years)*				
<50	74	26 (35.1%)	48 (64.9%)	0.669
≧50	68	22 (32.4%)	46 (67.6%)	
				
*Gender*				
Male	99	31 (31.3%)	68 (68.7%)	0.251
Female	43	17 (39.5%)	26 (60.5%)	
				
*Clinical stage*				
I–II	48	18 (37.5%)	30 (62.5%)	0.420
III–IV	94	30 (31.9%)	64 (68.1%)	
				
*T classification*				
T1–T2	100	39 (39.0%)	61 (61.0%)	0.011
T3–T4	42	9 (21.4%)	33 (78.6%)	
				
*N classification*				
N0–N1	63	21 (33.3%)	42 (66.4%)	0.897
N2–N3	79	27 (34.2%)	52 (65.8%)	
				
*Distant metastasis*				
Yes	4	2 (50.0%)	2 (50.0%)	0.423
No	138	46 (33.3%)	92 (66.7%)	

NPC, Nasopharyngeal carcinoma.

^*^χ^2^ test was applied to access the associations between miR-3188 expression and the clinicopathological parameters.
