# Author Correction: miR-3188 regulates nasopharyngeal carcinoma proliferation and chemosensitivity through a FOXO1-modulated positive feedback loop with mTOR–p-PI3K/AKT-c-JUN

**DOI:** 10.1038/s41467-021-22959-7

**Published:** 2021-05-14

**Authors:** Mengyang Zhao, Rongcheng Luo, Yiyi Liu, Linyuan Gao, Zhaojian Fu, Qiaofen Fu, Xiaojun Luo, Yiyu Chen, Xiaojie Deng, Zixi Liang, Xin Li, Chao Cheng, Zhen Liu, Weiyi Fang

**Affiliations:** 1 Cancer Center, Traditional Chinese Medicine-Integrated Hospital, Southern Medical University, Guangzhou, 510315 China; 2 Cancer Research Institute, Southern Medical University, Guangzhou, 510515 China; 3 Department of Pathology, Basic School of Guangzhou Medical University, Guangzhou, 510182 China

Correction to: *Nature Communications* 10.1038/ncomms11309, published online 20 April 2016.

This Article contains errors in Figure 5. In figure 5g the FOXO1 H&E image for HONE1-EBV cells is taken from a partially overlapping field of the Mock H&E image for HONE1-EBV cells. In addition, the FOXO1 immunohistochemistry image for FOXO1 HONE1-EBV cells is taken from a partially overlapping image of the FOXO1 immunohistochemistry image for FOXO1 SUNE1 cells. The incorrect image appears below.
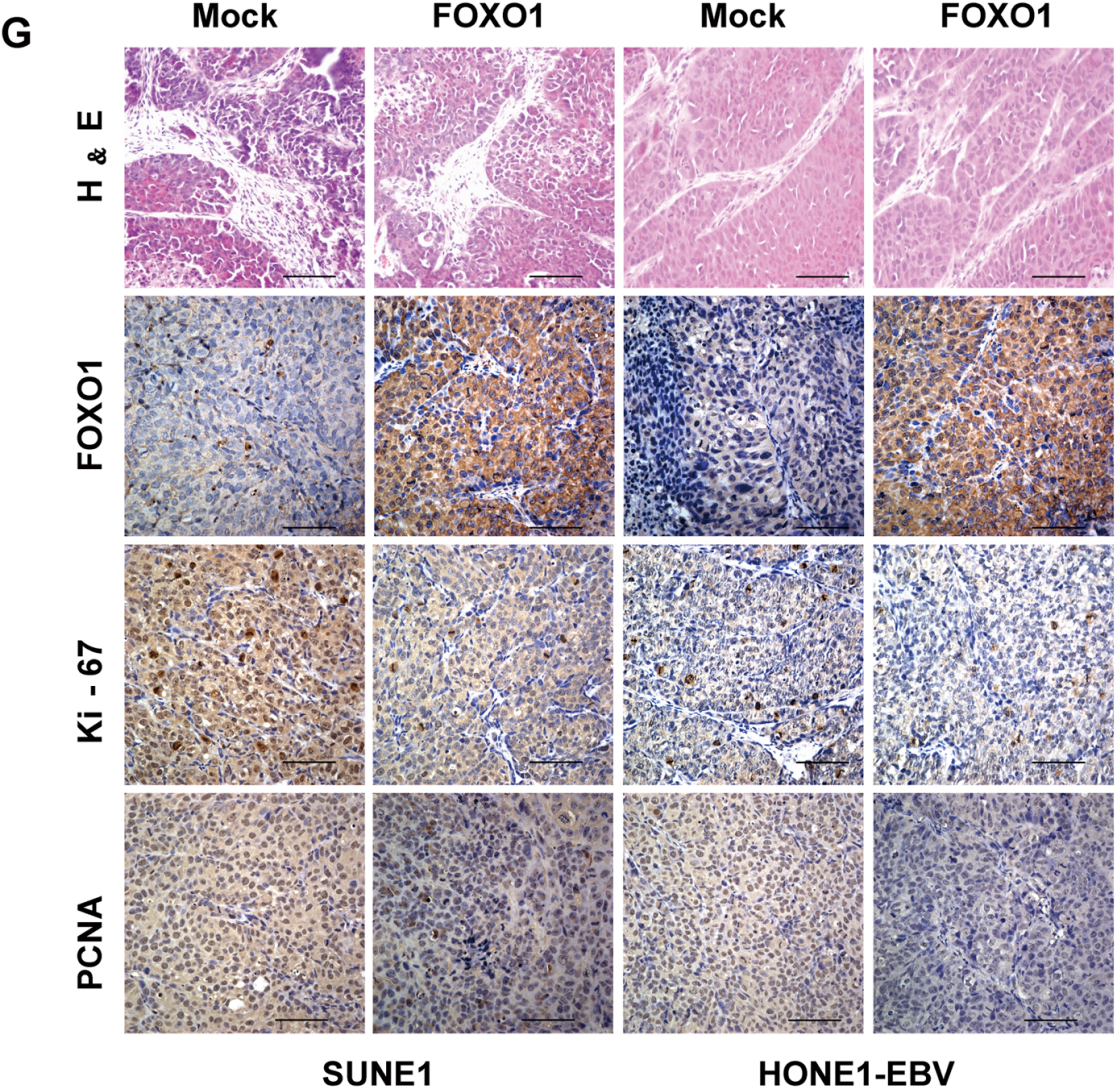


The correct image appears below.
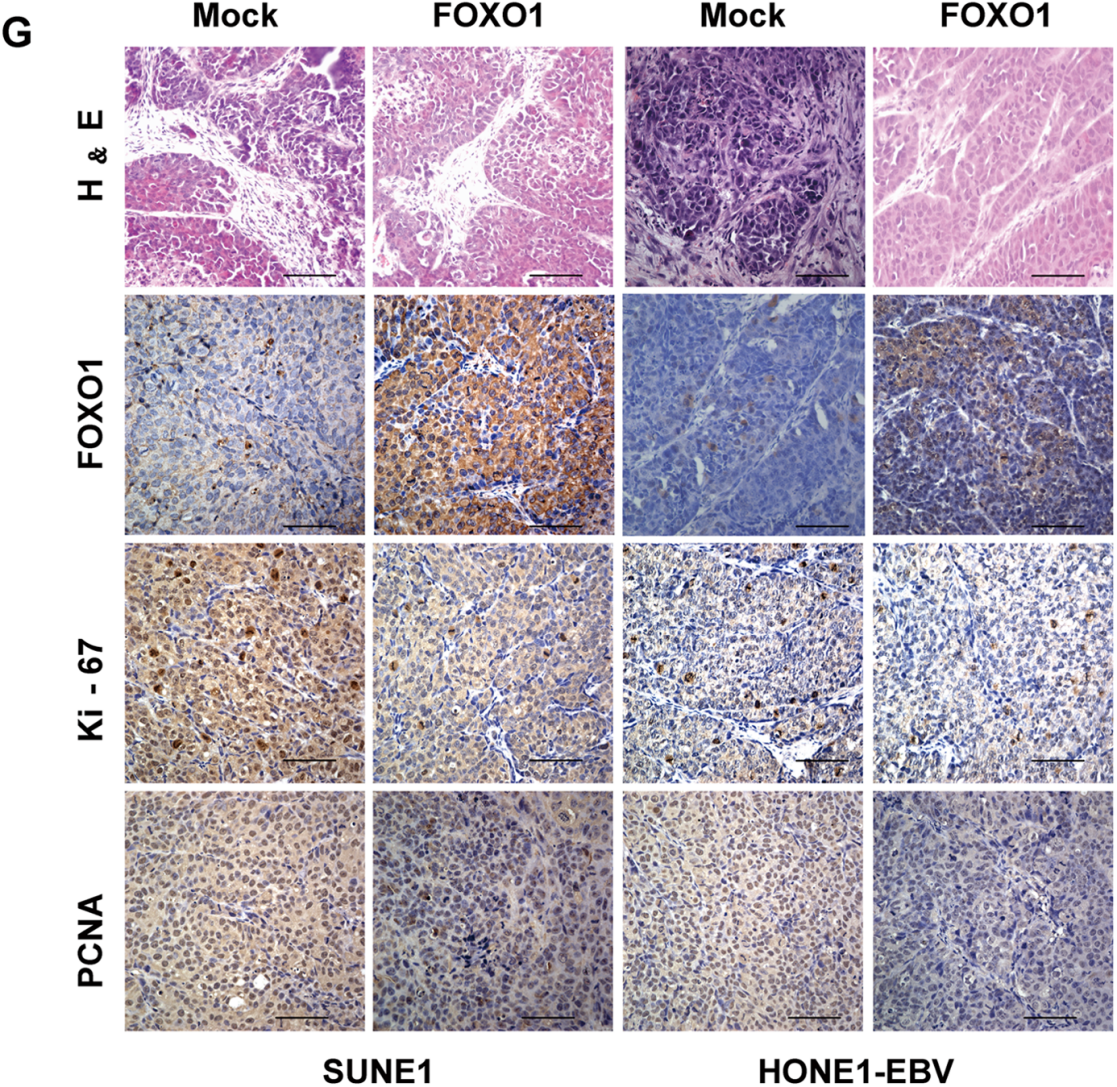


The PDF and HTML versions of the Article have not been corrected.

