# Supplementary material for: miR-3188 regulates nasopharyngeal carcinoma proliferation and chemosensitivity through a FOXO1-modulated positive feedback loop with mTOR–p-PI3K/AKT-c-JUN
Source: Nat Commun. 2016 Apr 20;7:11309. doi: 10.1038/ncomms11309 (PMC4842991; doi:10.1038/ncomms11309)
Supplement: Supplementary Information — Supplementary Figures 1-10 and Supplementary Tables 1-3 [file ncomms11309-s1.pdf]

Supplementary Information

Supplementary Figures

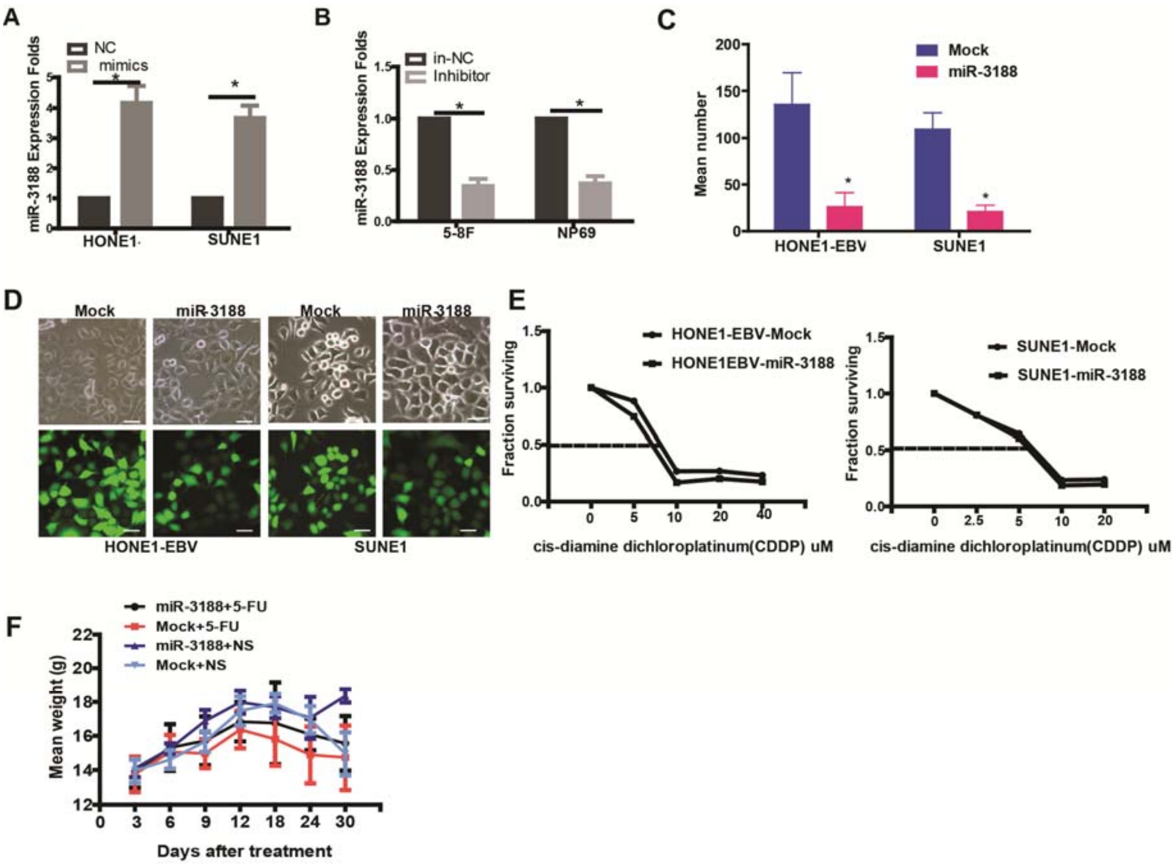

Supplementary Figure 1. Supplementary figures were related to Figure1. (A, B). RT-PCR showing transcriptional levels of the miR-3188 with U6 used as an internal control. Bar graph shows relative mRNA expression. Student's t-test, mean ± SD, **P* < 0.05. **(C).** NPC colony formation assays were performed after transfection with NC or miR-3188. Student's t-test, mean ± SD, **P* < 0.05. **(D).** HONE1-EBV and SUNE1 were transfected by lentiviruses containing LV-con or LV-miR-3188. Scale bar, 15 μm. **(E).** Dose-response curves of SUNE-1 and HONE1-EBV cells respectively treated with miR-mock or miR-3188 following 48 hrs treatment with DDP. **(F).** A fitted curve was used to determine the weight of each group.

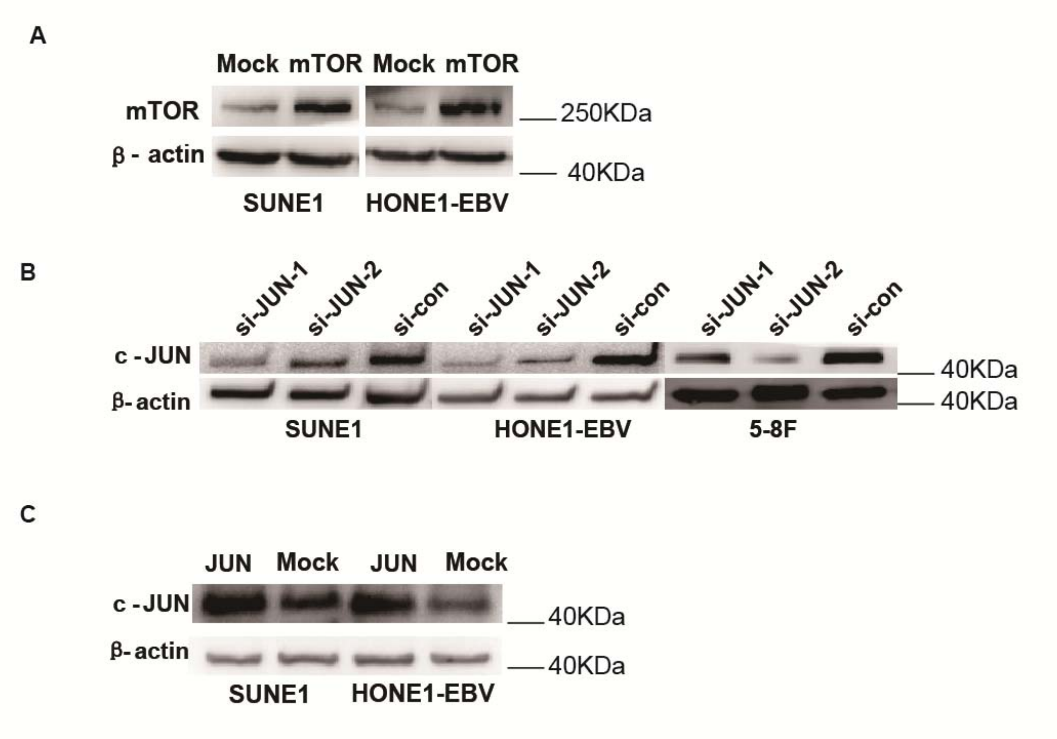

Supplementary Figure 2. The expression of mTOR and C-JUN were detected by western blot. (A). mTOR expression was examined by western blot in NPC cells transected with ectopic mTOR. **(B, C).** siRNAs and c-JUN plasmid were used to suppress or overexpress c-JUN expression. β-actin served as a loading control.

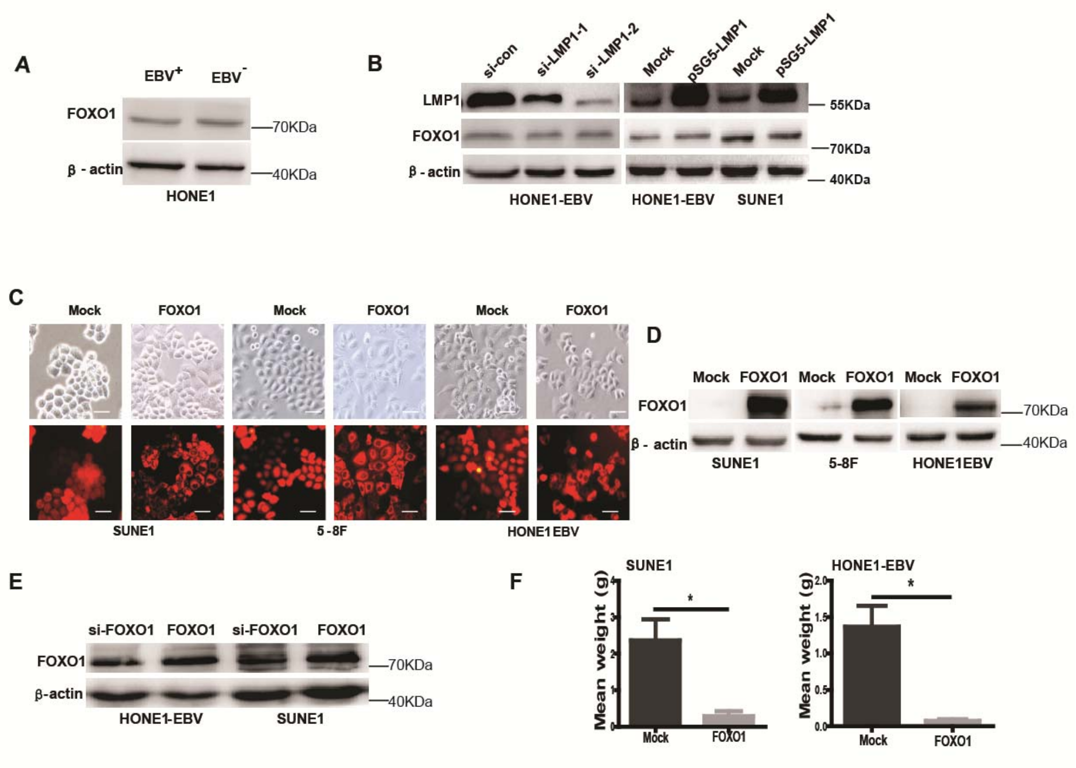

Supplementary Figure 3. Supplementary figures were related to Figure5. (A). Protein levels of FOXO1 in HONE1 and HONE1-EBV was measured by western blot. **(B).** Expression levels of LMP1 and FOXO1 were measured by western blot in NPC cells treated with si-con, siLMP1 or Mock, ectopic LMP1. β-actin served as a loading control. **(C).** HONE1-EBV, SUNE1 and 5-8F cells were transfected by lentiviruses containing LV-con or LV-FOXO1. Scale bar, 15 μm. **(D).** FOXO1 expression was detected after transfection with Mock and/or FOXO1. β-actin served as a loading control. **(E).** siRNA against FOXO1 as indicated in NPC cells by Western blot. β-actin served as a loading control. **(F).** Compared with mock cells, tumorigenicity of HONE1-EBV-FOXO1 and SUNE1-FOXO1 cells was markedly reduced *in vivo*. Student's t-test, mean ± SD, $*P < 0.05$.

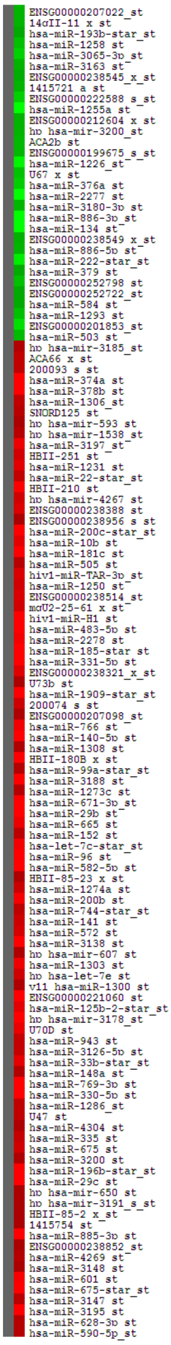

Supplementary Figure 4. miRNA expression profile microarray screening. Hierarchical cluster analysis of 82 miRNAs that were differentially expressed between SUNE1-FOXO1 and SUNE1-mock cells. Red represents upregulated miRNAs and green downregulated miRNAs. (GEO Accession Number: GSE78742).

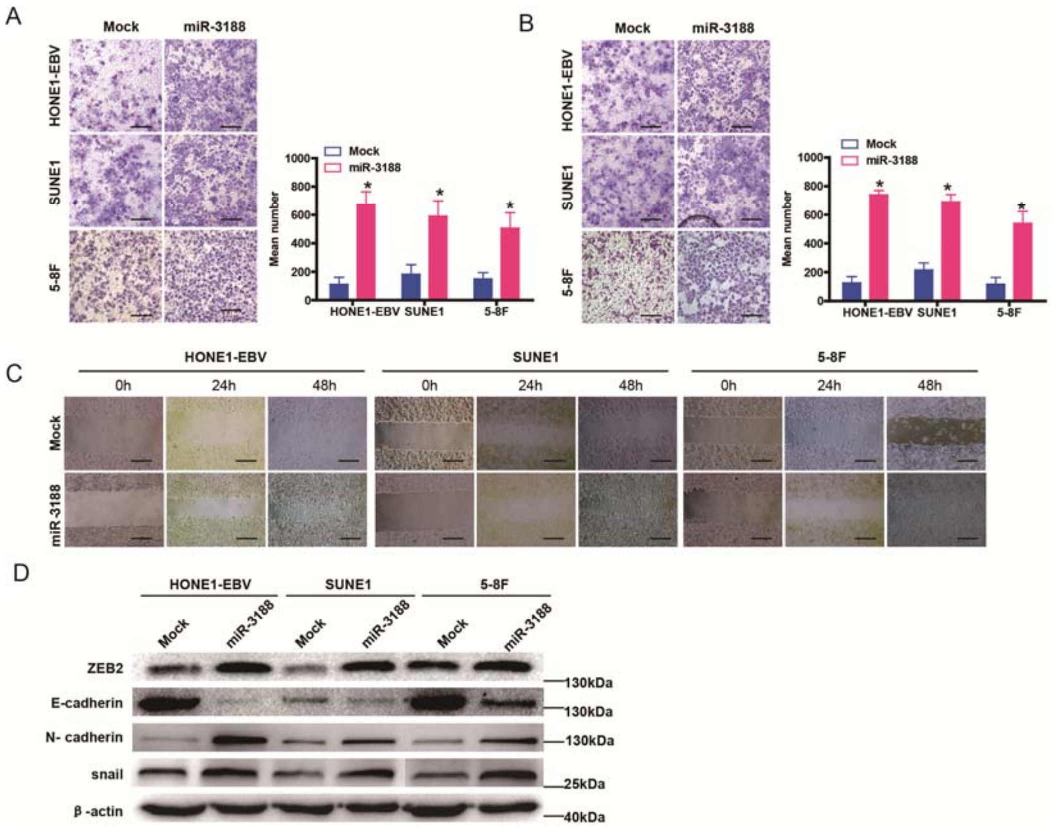

Supplementary Figure 5. miR-3188 promotes an epithelial-mesenchymal transition (EMT)-like process in NPC. Transwell assay (A), Boyden assay(B) and Wound healing assays (C) evaluating the migration of NPC cells. Scale bar, 30 μm, 60μm. Student's t-test, mean ± SD, $*P < 0.05$. (D). Western blotting detected the expression of key EMT-related factors including ZEB2, Snail, N-cadherin and E-cadherin. β-actin served as a loading control.

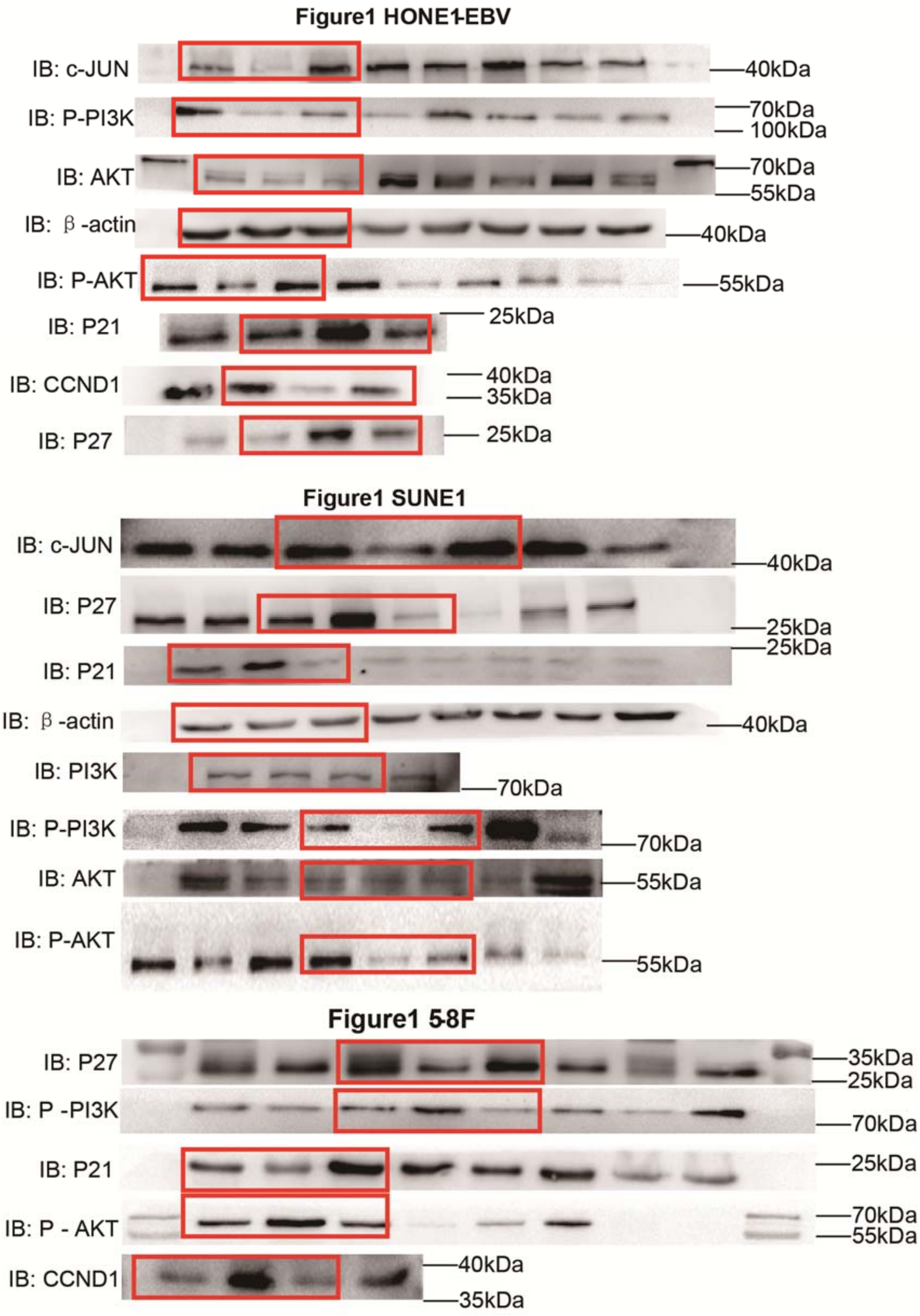

Supplementary Figure 6. Original Western blotting images of the Figure 1.

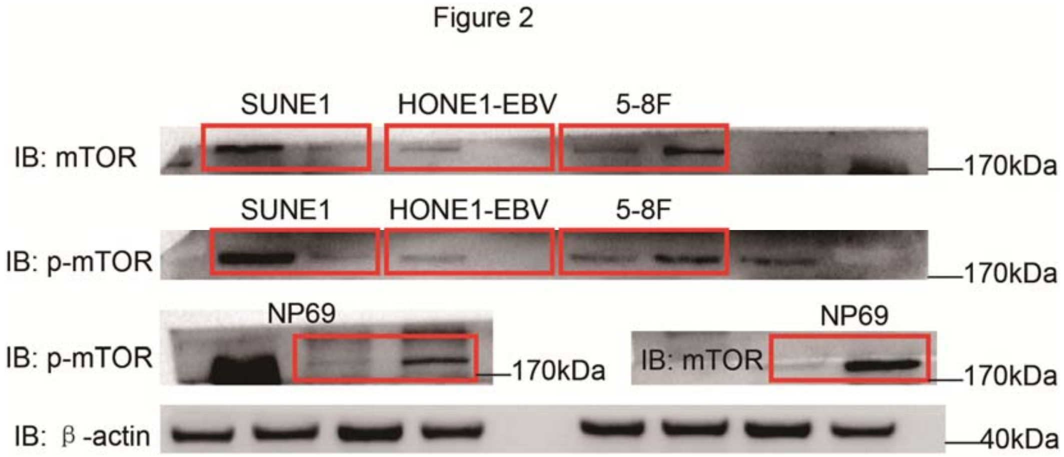

Supplementary Figure 7. Original Western blotting images of the Figure 2.

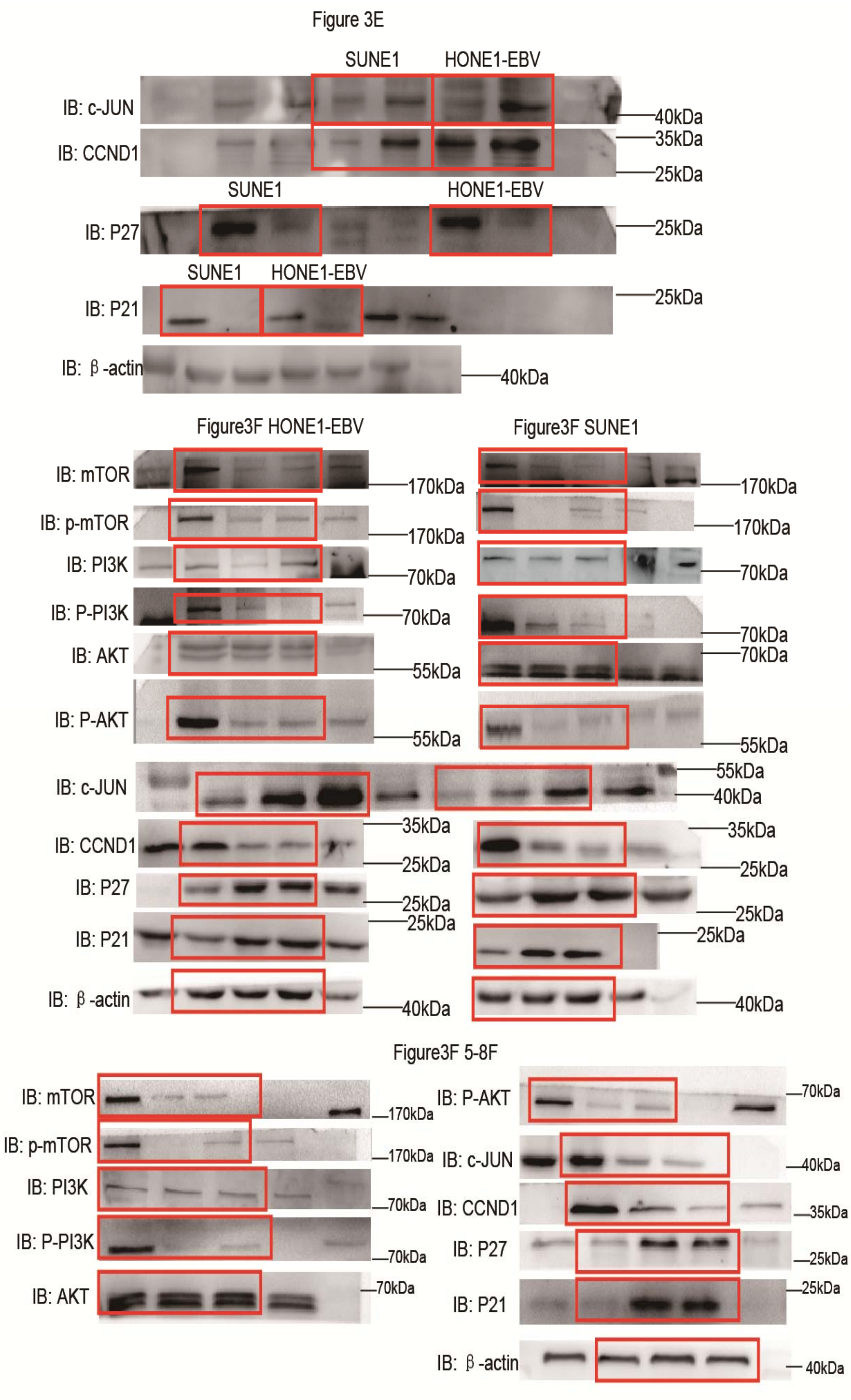

Supplementary Figure 8. Original Western blotting images of the Figure 3.

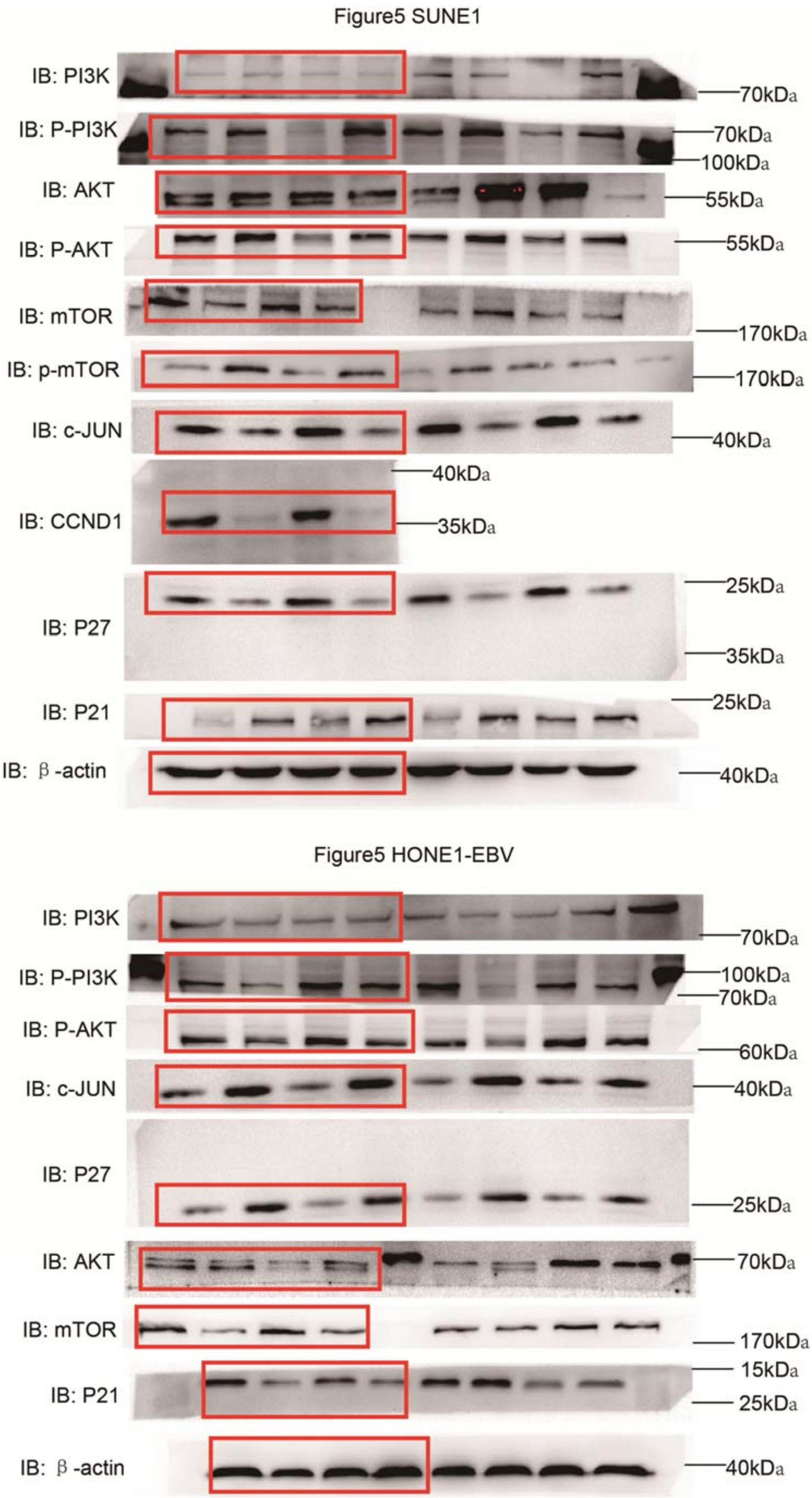

Supplementary Figure 9. Original Western blotting images of the Figure 5.

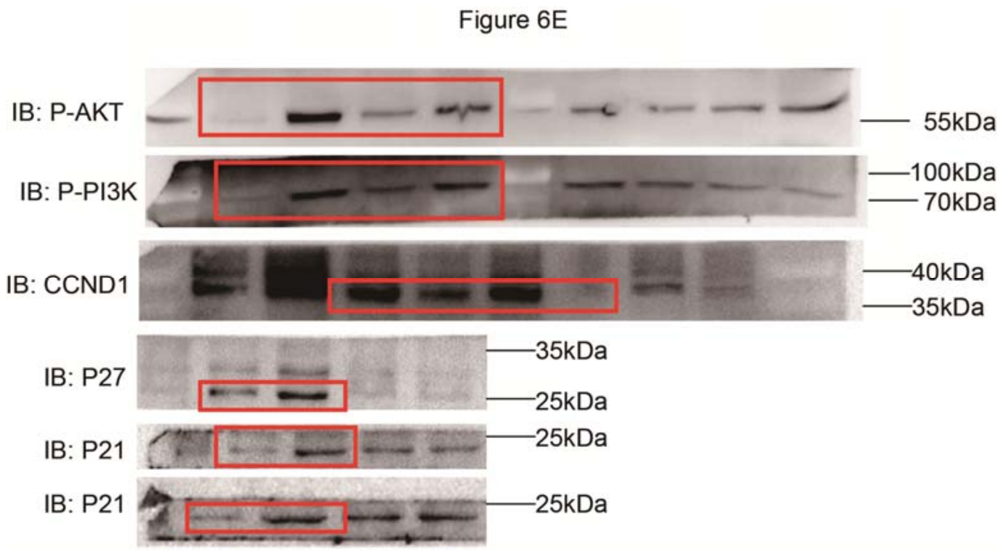

Supplementary Figure 10. Original Western blotting images of the Figure 6.

Supplementary Tables

Supplementary Table 1: The sequences used in this study.

gene	No.		sequence
c-JUN	1	Sense	5’ GGCACAGCUUAAACAGAAA dTdT 3’
		Antisense	3’ dTdT CCGUGUCGAAUUUGUCUUU 5’
	2	Sense	5’ CGCAGCAGUUGCAAACAUU dTdT 3’
		Antisense	3’ dTdT GCGUCGUCAACGUUUGUAA 5’
	3	Sense	5’ GACCUUAUGGCUACAGUAA dTdT 3’
		Antisense	3’ dTdT CUGGAAUACCGAUGUCAUU 5’
FOXO1	1	Sense	5’CUGCAUCCAUGGACAACAA dTdT 3’
		Antisense	3’ dTdTGACGUAGGUACCUGUUGUU 5’
	2	Sense	5’ CCAGAUGCCUAUACAAACA dTdT 3’
		Antisense	3’ dTdT GGUCUACGGAUAUGUUUGU 5’
	3	Sense	5’ CUCAAAUGCUAGUACUAUU dTdT 3’
		Antisense	3’ dTdT GAGUUUACGAUCAUGAUAA 5’
LMP1	1	Sense	5‘ CCUUGUCCUCUAUUCCUUU dTdT 3‘
		Antisense	3‘ dTdT GGAACAGGAGAUAAGGAAA 5‘
	2	Sense	5‘ GGAUCUACUUAUUGGAGAU dTdT 3‘
		Antisense	3‘ dTdT CCUAGAUGAAUAACCUCUA 5‘
	3	Sense	5‘ CCUUUGGCUCCUCCUGUUU dTdT 3‘
		Antisense	3‘ dTdT GGAAACCGAGGAGGACAAA 5‘
mTOR	1	Sense	5’-CCAAGGAGCUCCAGCACUATT-3’
		Antisense	5’-UAGUGCUGGAGCUCCUUGGTT-3’
	2	Sense	5’-GCAAAGACCUCAUGGGCUUTT-3’
		Antisense	5’-AAGCCCAUGAGGUCUUUGCTT-3’
	3	Sense	5’-CGGCAGAUCUGGUGGGAGATT-3’
		Antisense	5’-UCUCCCACCAGAUCUGCCGTT-3’
miR-3188 mimics		Sense	5‘AGAGGCUUUGUGCGGAUACGGGG3’
		Antisense	3‘UCUCCGAAACACGCCUAUGCCCC5'
Negative control		Sense	5’ UUCUCCGAACGUGUCACGUTT3’
		Antisense	3’ ACGUGACACGUUCGGAGAATT5’
miR-3188 inhibitor			5’CCCCGUAUCCGCACAAAGCCUCU3'
Inhibitor negative control			5’UUUGUACUACACAAAAGUACUG 3’
EMSA probe sequence mut A			TCCGTGTTCCGTTATCTGAGGGTCTTC
EMSA probe sequence mut B			ACTCAGGCTCTCGAAGCGCGCGTCCTTC
EMSA probe sequence mut C			GAGTTAGCTATCGAAGGCAGCAGGGCC

Supplementary Table 2: The primers used in this study.

Primers name		Sequence (5'-3')
FOXO1	Forward	AAGGCCATCGAGAGCTCGGC
	Reverse	GCTCGGCTTCGGCTCTTAGCA
c-JUN	Forward	CTGCGTCTTAGGCTTCTCC
	Reverse	CTCGCCCAAGTTCAACAA
LMP1	Forward	CGTTATGAGTGACTGGACTGGA
	Reverse	TGAACAGCACAATTCCAAGG
mTOR	Forward	AAGCCGCGCGAACCTC
	Reverse	GGCATCTGAGCTGGAAACCA
ARF5	Forward	ATCTGTTTCACAGTCTGGGACG
	Reverse	CCTGCTTGTTGGCAAATACC
MiR3188		AGAGGCTTTGTGCGGATACGGG
U6		CTCGCTTCGGCAGCACATATA
luciferase-mTOR	Forward	ATCGCTCGAGGACTTAACTCACAAGAGAACTC
	Reverse	ATCGCGGCCGCCAACATGGTGTCTAGACATGG
luciferase-miR-3188	Forward	ATCGCGGCAGCCGCTGTTGACGTGGC
	Reverse	ATCGCGTACCAAGCAGAGCATAGCACTGT
c-JUN -A	Forward	GGCAGCCGCTGTTGACGTGGC
	Reverse	CGCTGCCGAACCTGTGCCCTTC
c-JUN -B	Forward	GTGACCAGCCCGTTGGACTGGA
	Reverse	GATGGAAACACCCCTTCTACATGA
c-JUN-C	Forward	AGGGCTGTGATGTCCTGGGCGAGG
	Reverse	GTACCAAGCAGAGCATAGCACTGT
EMSA-c-JUN-WT	Forward	GAGCGGATAACAATTTCACACAGG
	Reverse	AACACAGCACCTCTTTTTGT
EMSA-c-JUN-mut-A	Forward	ACAAAAAGAGGTGCTGTGTT
	Reverse	AACGACGGCCAGTGCCAAGC
EMSA-c-JUN-mut-B	Forward	GAGCGGATAACAATTTCACACAGG
	Reverse	AACACAGCACCTCTTTTTGT
EMSA-c-JUN-mut-C	Forward	ACAAAAAGAGGTGCTGTGTT
	Reverse	AACGACGGCCAGTGCCAAGC

Supplementary Table 3. A list of antibodies used for Western blot, IHC staining, CHIP, EMSA

Name of anti-body	Cat.No	Company	Mol weight	Dilution (WB/IHC/CHIP/EMSA)
c-JUN	9165	CST	43,48 kDa	1:1000 (WB); 1:50 (CHIP);1:50 (EMSA)
c-JUN	24909-1-AP	PTG	39 kDa	1: 250 (IHC)
FOXO1	ab52857	abcam	80 kDa	1:250 (IHC)
FOXO1	2880	CST	78-82 kDa	1:1000 (WB)
LMP1	HPA002933	Sigma-Aldrich	63 kDa	1:1000 (WB)
P-AKT	4060	CST	60kDa	1:1000 (WB)
P-PI3K	4228	CST	85 kDa	1:1000 (WB)
AKT	4691	CST	60kDa	1:1000 (WB)
P27	25614-1-AP	PTG	27 kDa	1:500 (WB)
snail	3895	CST	29 kDa	1:1000 (WB)
c-fos	ab134122	abcam	62 kDa	1:1000 (WB)
CCND1	ab134175	abcam	34 kDa	1:1000 (WB)
P21	2947	CST	21 kDa	1:1000 (WB)
PCNA	10205-2-AP	PTG	36-38 kDa	1:30 (IHC)
N-cadherin	4061	CST	140 kDa	1:1000 (WB)
Ki67	Ab16667	abcam	-----	1:100 (IHC)
PI3K	60225-1-Ig	PTG	85 kDa	1:500 (WB)
E-cadherin	3195	CST	135kDa	1:1000 (WB)
p-mTOR	5536	CST	289 kDa	1:1000 (WB)
mTOR	04-385	millipore	289 kDa	1:1000 (WB); 1:250 (IHC)
β-actin	sc-1616	Santa	43 kDa	1:1000 (WB)
ZEB2	sc-271984	Santa	157 kDa	1:1000 (WB)
